# A variety-specific analysis of climate change effects on California winegrapes

**DOI:** 10.1007/s00484-024-02684-8

**Published:** 2024-04-23

**Authors:** Lauren E. Parker, Ning Zhang, John T. Abatzoglou, Isaya Kisekka, Andrew J. McElrone, Steven M. Ostoja

**Affiliations:** 1 USDA California Climate Hub, Davis, CA 95616 USA; 2 Institute of the Environment, University of California Davis, Davis, CA 95616 USA; 3 Department of Land, Air and Water Resources, University of California Davis, Davis, CA 95616 USA; 4 Department of Management of Complex Systems, University of California Merced, Merced, CA 95343 USA; 5 Department of Biological and Agricultural Engineering, University of California, Davis, 95616 USA; 6 USDA-ARS Crops Pathology and Genetics Research Unit, Davis, CA 95616 USA; 7 Department of Viticulture and Enology, University of California Davis, Davis, CA 95616 USA; 8 USDA-ARS Sustainable Agricultural Water Systems Research Unit, Davis, CA 95616 USA

**Keywords:** Agroclimatic metrics, California, Climate change, Phenology, Winegrapes

## Abstract

California contains a broad geography over which climate conditions can be suitable for cultivating multiple varieties of winegrapes. However, climate change is projected to make winegrape cultivation more challenging across many of California’s winegrowing regions. In order to understand the potential effects of climate change on winegrapes, this study models variety-specific phenology for six winegrape varieties and quantifies the change in phenology and viticulturally-important agroclimate metrics over 12 of California’s American Viticultural Areas (AVAs) by the mid-21st century. Results show more rapid development for winegrapes with earlier budburst, flowering, veraison, and maturation across all varieties and AVAs. Cabernet Sauvignon shows the greatest change in phenology timing, while Chardonnay shows the least change. Likewise, the West Sonoma Coast AVA shows the greatest average change in phenology timing across varieties and development stages and Lodi AVA shows the least. Projected changes in agroclimatic metrics include an additional month of potentially damaging heat days (above 35 °C) in some AVAs, and decreases in frost days. These results have implications for numerous factors related to viticultural production, including water resources management and crop yield and quality, and underscore the need for California winegrape growers to improve their resilience to climate change by adopting strategies such as increasing soil health and water use efficiency and selecting cultivars suited for future climate conditions. By conducting climate effects analyses at the variety-specific and AVA scale, important information is provided to the winegrowing industry at a resolution that can support decision-making towards resilience.

**Supplementary Information:**

The online version contains supplementary material available at 10.1007/s00484-024-02684-8.

## Introduction

Winegrapes (*Vitis vinifera*) are thought to have been first cultivated from wild relatives in southwest Asia as early as 7000 BCE (Bouby et al. 2013). Winegrape cultivation in California began in earnest in the early 19th century (Anderson et al. 2003) and as of 2022, California grows winegrapes on more than 2,428 square kilometers, yielding approximately 3.4 million crushed tons (CDFA 2022; CDFA 2023). This scale of viticulture and the accompanying wine production contributes > 400,000 jobs and >$70 billion to the state economy and makes California the fourth largest wine producing region globally (CWI, 2022).

California’s Mediterranean macroclimate and diverse mesoclimates, ranging from cool and coastal to warm and inland, offer a broad geography over which climate conditions can be suitable for cultivating many varieties of winegrapes. However, in California, climate change is projected to increase temperature, interannual precipitation variability, and the frequency and intensity of extreme heat events, as well as exacerbate drought and alter pest and disease pressure (Pathak et al. 2018). Unsurprisingly, climate change is projected to affect winegrape yield and quality across many of California’s winegrowing regions (Hannah et al. 2013). To this point, projected increases in extreme heat exposure could slow grapevine development, reduce berry weight, negatively impact berry quality, and alter the chemical composition of winegrapes and subsequently the characteristics of wine (Parker et al. 2020a). Similarly, multiple studies have shown that climate change, and extreme heat in particular, may reduce yields and suitable growing regions for wine in California (Diffenbaugh et al. 2011; Monteverde and De Sales 2020; White et al. 2006).

Despite viticulture’s well-known sensitivity to climate, the diversity of winegrape varieties allows for grape production across a wide range of climates around the world (Jones 2015; Jones and Webb 2010). In fact, it has been argued that varietal variation could provide an adaptive opportunity to climate change and potentially reduce the projected contraction of suitable growing locations among today’s wine regions; this argument is predicated on the variation in physiological tolerances and phenology across winegrape varieties (Morales-Castilla et al. 2020; Wolkovich et al. 2017). Climatic tolerances vary not only across varieties but also throughout the growing season. For winegrapes, changes in the timing of phenological development can influence berry size, color, chemistry and wine quality; however, by matching the climate conditions to variety-specific phenology and climatic tolerances, growers can produce characteristic winegrapes (Parker et al. 2020b).

Previous studies have shown that the effects of climate change on winegrapes and grape phenology will vary by location and variety (e.g., Ausseil et al. 2021; Hannah et al. 2013; Webb et al. 2007), and understanding potential climate risks at local scales is useful for management planning (e.g., Babin et al., 2022). The objective of this study is to explore the potential effects of projected climatic change on winegrape production in California across multiple winegrape-growing regions known as American Viticultural Areas (AVAs) and across multiple winegrape varieties. To do this, we model potential shifts in variety-specific phenology and quantify the change in viticulturally-important climate metrics at the AVA scale. Given the economic importance of California viticulture and the climate changes expected, improving the understanding of how climate change may affect winegrape production across growing regions and varieties will assist winegrape growers and the broader wine industry in identifying and prioritizing adaptation actions to meet location- and variety-specific climate-mediated challenges.

## Data and methods

### Data

#### Climatological data

Daily maximum and minimum temperature (Tx, Tn), precipitation (Pr), and reference evapotranspiration (ETo) for the contemporary (1991–2020) period were obtained from the 4-km gridded dataset (gridMET, https://www.climatologylab.org/gridmet.html) of Abatzoglou (2013). gridMET is a spatially-continuous, daily dataset of surface meteorological conditions developed using the PRISM dataset of PRISM Climate Group, Oregon State University (https://prism.oregonstate.edu, see also Daly et al. 1997 and Daly et al. 2008) and regional reanalysis data (NLDAS-2, see Xia et al. 2012) covering the contiguous United States from 1979 to present (Abatzoglou 2013). Daily Tx, Tn, Pr, and ETo were also acquired for 20 global climate models (GCMs, Table 1) participating in the fifth coupled model intercomparison project (CMIP5) for the mid-21st century (2040–2069) period for representative concentration pathway (RCP) 4.5. RCP 4.5 represents a future scenario in which greenhouse gas emissions peak in the early-mid 21st century and then decline, resulting in moderate warming. These GCM data were obtained from the 4-km Multivariate Adaptive Constructed Analogs (MACA, https://www.climatologylab.org/maca.html) dataset of Abatzoglou and Brown (2012). MACA is a statistical downscaling method that applies a constructed analog approach for mapping daily GCM data to observed data (Abatzoglou and Brown 2012). The approach further applies bias correction using an equidistant quantile mapping methodology (Li et al. 2010). Here, we use MACA trained using gridMET data which provides interoperability between the historical observed data and future projections. For both gridMET and MACA, ETo is calculated for a well-watered grass surface using the Penman-Monteith method (Allen et al. 1998; Walter et al., 2000). These datasets were chosen because they provide a spatially continuous and temporally complete record of climate conditions suitable for local and landscape-scale agricultural research. RCPs were used rather than the newer Shared Socioeconomic Pathways (SSPs) used in the current sixth coupled model intercomparison project (CMIP6) because downscaled SSP data are currently limited. Studies have generally shown similarities between climate models participating in the CMIP5 and CMIP6 over California (Krantz et al., 2021) and broadly similar hydroclimatic changes (Cook et al., 2021). While other RCP scenarios exist, we focus our results on RCP 4.5 because it provides a more conservative measure of potential change; we also note that the variability between models exceeds the variability between RCPs at mid-century time horizons (Kharin et al. 2013). Elevation data were acquired from the digital elevation model (DEM) associated with the gridMET dataset.

**Table 1:** Global Climate Models participating in the 5th Coupled Model Intercomparison Project (CMIP5) used in this study

GCM	Country of Origin
bcc-csm1-1	China
bcc-csm1-1-m	China
BNU-ESM	China
CanESM2	Canada
CCSM4	USA
CNRM-CM5	France
CSIRO-Mk3-6-0	Australia
GFDL-ESM2M	USA
GFDL-ESM2G	USA
HadGEM2-CC365	United Kingdom
HadGEM2-ES365	United Kingdom
inmcm4	Russia
IPSL-CM5A-LR	France
IPSL-CM5A-MR	France
IPSL-CM5B-LR	France
MIROC5	Japan
MIRCO-ESM	Japan
MIROC-ESM-CHEM	Japan
MRI-CGCM3	Japan
NorESM1-M	Norway

#### Winegrape data

Six winegrape varieties – 3 red varieties and 3 white varieties – were selected for analysis based on a combination of economic importance, acreage, and variety-specific information available in the literature for developing phenology models and environmental tolerance thresholds. The red winegrape varieties selected include Cabernet Sauvignon, Pinot Noir, and Zinfandel, and the white winegrape varieties selected are Chardonnay, Pinot Gris, and Sauvignon Blanc. As of 2022, these six varieties comprise the top three red and top three white winegrape varieties by dollar value, and collectively these six varieties comprise more than 70% of the total dollar value of winegrapes sold in California (CDFA, 2023).

#### American viticultural area data

**Fig. 1:**
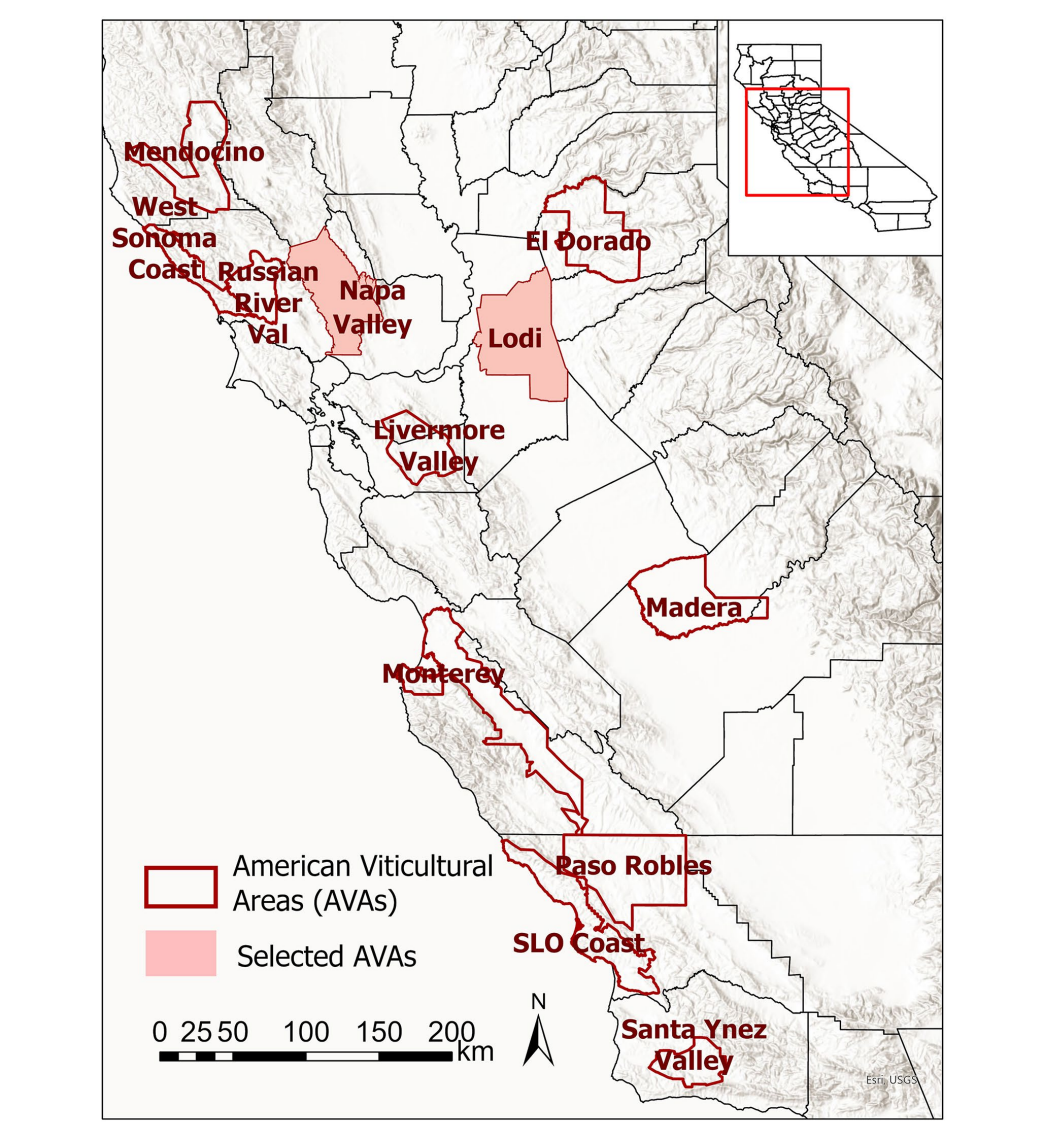
The 12 American Viticultural Areas (AVAs) explored in this analysis are outlined in red. Two AVAs selected to illustrate within-AVA spatial variability of results are shaded in pink. For reference, California counties are represented on the map by thin, grey lines

Twelve American Viticultural Areas (AVAs) within California were chosen as representative of a range of climate conditions that can characterize climate change effects broadly. The selected AVAs provide a range of mesoclimates, are similar in size, represent both heterogeneous and homogenous topography, and cover both coastal and inland locations as well as Northern and Southern California locations. The relative importance of the AVAs to statewide winegrape production was also considered; although production acreage, crushed tons, and value are calculated at the crush district scale, which is a larger spatial scale than the AVA, discussion with industry experts helped to identify key AVAs. The 12 AVAs are: El Dorado, Livermore Valley, Lodi, Madera, Mendocino, Monterey, Napa Valley, Paso Robles, Russian River Valley, Santa Ynez Valley, San Luis Obispo (SLO) Coast, and West Sonoma Coast (Fig. 1). AVA shapefiles were downloaded from the U.S. Department of Treasury Alcohol and Tobacco Tax Trade Bureau’s AVA Map Explorer (https://www.ttb.gov/images/AVA/). A grid cell was considered as part of an AVA if its center was located within the AVA shapefile.

### Variety-specific phenology modeling

Winegrape development is a complex, multi-phasic cycle that has been simplified for our assessments of climate effects on variety-specific phenology and exposure. Following Parker and Abatzoglou (2018), we developed models for each variety using published climatic thresholds (Table 2); models assessed cold hardiness and changes in the timing of chill completion, budburst, flowering, veraison, and maturity.

**Table 2:** Variety-specific thresholds used to assess the changes in the timing of chill completion, budburst, flowering, veraison, and maturity. All DDc values are from Ferguson et al. (2014); GDD for budburst, flowering and veraison are from Zapata et al. (2017); and BEDD for maturity are from Gladstones (1992). For definitions of DDc and GDD, refer to Table 3; for a definition of BEDD, refer to the manuscript subsection on *Maturity*

Grape Variety	Chill	Budburst		Flowering		Veraison		Maturity
	DD_C_	GDD	T_base_ ℃	GDD	T_base_ ℃	GDD	T_base_ ℃	BEDD
Cabernet Sauvignon	700	101	8.3	240	10.4	598	12.5	1300
Pinot Noir	300	79	8.1	256	9.7	578	12.1	1150
Zinfandel	500	132	7.2	294	9.1	639	11.2	1200
Chardonnay	600	114	6.5	354	8.2	727	9.7	1150
Pinot Gris	400	130	6.9	313	8.3	678	10.4	1100
Sauvignon Blanc	300	101	7.4	327	8.8	607	11.1	1150

#### Chill accumulation

Sufficient time exposed to cool temperatures, known as “chill,” is necessary for fruit development and good yields in many perennial crops (Luedeling et al. 2009), including winegrapes. There are multiple models for calculating chill accumulation; here we calculate chilling degree days (DD_C_) using hourly temperature (T) and we use a base temperature (T_base_) of 10 °C for all six varieties accumulating from November to February, after Ferguson et al. (2014).

#### Budburst, flowering, and veraison

As vineyards are exposed to warmer temperatures in spring they begin to bloom. Budburst marks the first major stage in winegrape development and is reached when 50% of dormant buds show green tissue (Zapata et al. 2017). Flowering is the second major stage of winegrape development; it is reached when 50% of flower caps have dropped (Zapata et al. 2017). With further heat accumulation, fruit begins to develop and winegrapes will change color during a development phase known as veraison. Budburst, flowering, and veraison occur when growing degree day (GDD) accumulation reaches the defined threshold for the given stage. GDD are units of heat accumulation commonly used to track the development of crops during the growing season. For each of these stages, GDD accumulation is calculated following Zapata et al. (2017) using variety- and stage-specific T_base_. Although the base temperatures provided in Zapata et al. (2017) are based on a January 1 start date for GDD accumulation, prior experiments showed this approach to result in poor model performance over our areas of interest, with particularly early development modeled at lower latitudes with warmer winter and spring temperatures. To address this, we do not begin GDD accumulation until daylength > 11-hours based on the growing season index (Jolly et al. 2005); this modification follows latitude or daylength adjustments in other heat summation models in viticulture (e.g., Gladstones 1992; Huglin 1978).

#### Maturity

Fruit development continues through summer, and when sufficient heat accumulation has occurred the winegrapes reach maturity. Winegrape harvest dates are influenced by non-climatic conditions such as grape chemistry preferences (e.g., brix and acid levels within the fruit); here we estimate winegrape maturity by applying a threshold for the biologically effective degree days (BEDD) needed to reach maturation (Gladstones 1992). BEDD incorporates two factors: one for adjusting the diurnal temperature range and another for correcting day length. The diurnal temperature range (DTR) is the difference between daily maximum and minimum temperatures, and when calculating BEDD the DTR factor increases when the DTR exceeds 13 °C and decreases when it falls below 10 °C. The day length correction factor ranges from 1.00 at 40° latitude to 1.045 at 50° latitude. For detailed calculations, please refer to Hall and Jones (2010). Here we begin BEDD accumulation on April 1. BEDD were used to determine the timing of maturity due to a lack of threshold information for GDD or other indices for maturity (e.g., the Huglin Index) in the literature for all our selected varieties. Our maturity analysis is not limited by established minimum and maximum BEDD values for quality winegrape production (Jones et al. 2010) as experiments showed all of our selected AVAs maintain BEDD within the bounds for production under both contemporary and future climates.

### Quantifying changes in agroclimatic metrics

Quantifying agriculturally-relevant climate metrics offers a picture of physiologically-important environmental conditions, and monitoring these metrics for changes over time can be useful for identifying potential adaptation needs within cropping systems. We selected 14 metrics of importance to viticulture (Table 3). For the majority of the metrics, we take a generalized approach to calculations and we do not use any variety- or stage-specific values for T_base_. However, for cold hardiness and frost damage, we use variety- and stage-specific thresholds, respectively.

**Table 3:** 14 Agroclimatic metrics selected for this analysis, their calculation, and their relevance to viticulture

Agroclimate Metric	Calculation	Relevance to Viticulture
Growing Degree Days (GDD)	GDD are calculated following Zapata et al. (2017) using T_lower_ = 10 °C and T_upper_ = 32 °C. GDD over the growing season is accumulated from April to October.	GDD are used in phenology models to estimate the timing of crop (and/or pest) development, which can be useful for in-season planning. Increased GDD can speed up crop development.
Cold Hardiness (H_ini_)	Cold hardiness is calculated as the number of days between September 21 and December 21 with temperatures below variety-specific thresholds (Ferguson et al. 2014): *Cabernet Sauvignon*: -10.3℃ *Pinot Noir*: -11.5℃ *Zinfandel*: -10.4℃ *Chardonnay*: -11.8℃ *Pinot Gris*: -12.0℃ *Sauvignon Blanc*: -10.6℃	Cold hardiness is the minimum overwinter temperature that can be tolerated without damage. As winter progresses, vines become increasingly cold tolerant. Here we use the cold tolerance thresholds associated with the fall season to provide a conservative view of potential exposure.
Chilling Degree Days (DD_C_)	Chill accumulation is calculated as DD_C_ using hourly T and T_base_ = 10 °C accumulating from November to February after Ferguson et al. (2014).	Chill is required in order for vines to fully break bud. Insufficient chill can lead to erratic budbreak and can impact fruit set and yields. Warmer winter and spring temperatures can reduce or delay chill accumulation.
Frost Damage Days (FDD)	FDD is calculated as the total number of days per year with T_n_ below the following stage-specific frost damage thresholds (Jones 2003): *Sap Bleeding*: -2.5 °C; *Budburst*: -2.2 °C; *Flowering*: -0.5 °C; *Veraison* and *Maturation*: 0 °C	Spring frost can damage buds and young shoots, resulting in uneven ripening and declines in yield. Fall frosts can cause defoliation of the vines prior to harvest, risking sun scald.
Last Spring Freeze (LSF)	The last day of the calendar year prior to June 30 with T_n_ ≤ 0 °C.	LSF is an important consideration for early-blooming and frost-sensitive perennials. Earlier LSF can also increase pest pressure.
First Fall Freeze (FFF)	The first day of the calendar year commencing July 1 with T_n_ ≤ 0 °C.	FFF is important for late-maturing varieties that may suffer damage from a fall freeze.
Freeze-Free Season (FFS)	The difference – in number of days – between the LSF and FFF (FFF [minus] LSF).	A certain length of FFS is required for vines to complete their annual development cycle. The FFS can inform the geography of cultivation. Longer FFS can also increase pest pressure.
Hot Days (HD)	The number of days with T_x_ >35 °C (Jones 2015) during the growing season (April – October).	Hot days can negatively affect grape development and yield (White et al. 2006). Impacts are dependent on HD timing and vine heat tolerance.
Heatwaves (HW)	Heatwave events are defined as 3 + consecutive days (Gershunov et al. 2009; Sheridan and Lee 2018) with T_x_ > 98th percentile of 1991–2020 summer (June-August) T_x_.	Consecutive days with high temperatures can impact grape yield and quality, depending on the timing and absolute temperatures (Martínez-Lüscher et al. 2020). Additionally, heatwaves have implications for vineyard worker safety and productivity.
Diurnal Temperature Range (DTR)	DTR is the difference between daily T_x_ and T_n_. We calculate DTR over August – October.	Reduced DTR can speed berry development and alter berry chemistry (Cohen et al. 2012a, b), though effects can vary by cultivar (Jones et al. 2012)
Diurnal Temperature Range > 20 °C (DTR20)	The number of days with DTR > 20 °C (White et al. 2006) over August to October.	High DTR in cool climate regions keeps malic acid and acidity in grapes and wine, while low DTR in warm regions makes grapes and wine more fruity and less acidic (Gladstones 1992).
Excess Precipitation Days (Pr_ex_)	The number of days with precipitation > 5 mm (Mosedale et al. 2015) during May to October.	Dry weather from May to October is preferred for optimum photosynthesis, ripening and balance. Excess precipitation during this period can impact bloom, promote diseases, and dilute berries (Jones 2003).
Winter Accumulated Precipitation (Pr_acc_)	Total accumulated precipitation in mm from November of the previous year to January of the current year.	Winter precipitation accumulation is necessary for soil moisture recharge (Jones 2003).
Crop Evapotranspiration (ET_c_)	ET_c_ is the adjusted reference ET (ET_o_) using the crop coefficient (K_c_) (ET_c_ = ET_o_*K_c_). The total ET_c_ is accumulated over the growing season (April – October) with units in mm.	ET_c_ is commonly used in irrigation models and decision support systems (Zhang et al. 2021). Changes in ET_c_ can indicate changes in irrigation demand.

For each metric, we calculated both the annual value and the 30-year average value over the contemporary period and the future period at the scale of the 4-km gridMET and MACA data. These calculations were done for each of 20 GCMs and the 20-model mean values were computed to represent the average condition of the future period. The grid cell values were then averaged across each AVA, providing a measure of the metric at the AVA scale and at the annual and climatological time step. The difference between 30-year average values for the future and contemporary periods were calculated (future [minus] contemporary); temporal trends in each metric were also assessed for each AVA over each 30-year period using a Theil-Sen estimator, and a Mann-Kendall test was applied to determine the significance of the trend (α = 0.05) with a null hypothesis of no significant trend. Finally, a supplemental analysis explored correlations between both phenology and agroclimatic metrics and annual mean temperature and elevation.

## Results

Across all varieties and AVAs, we show that phenology shifts towards later chill completion and earlier budburst, flowering, veraison, and maturation. While the between-variety difference in phenology changes is not large, Cabernet Sauvignon consistently shows the greatest change in phenology timing between the contemporary and future periods, while Chardonnay shows the least change. The between-AVA difference in phenology change is more pronounced with the West Sonoma Coast showing the greatest change in phenology timing across development stages and Lodi showing the least. Beyond phenology, results also show that climate change increases the incidence of some potentially damaging events, such as days above 35 °C and heatwaves, while decreasing the incidence of others, such as frost days. As with phenology, geography influences the degree of change projected for these and other agroclimatic metrics. Below we detail the results of our analysis, presenting relationships between phenology and variety, AVA, and geography, and the projected changes in viticulturally-important agroclimatic metrics at the AVA scale.

### Changes in phenology by variety

**Fig. 2:**
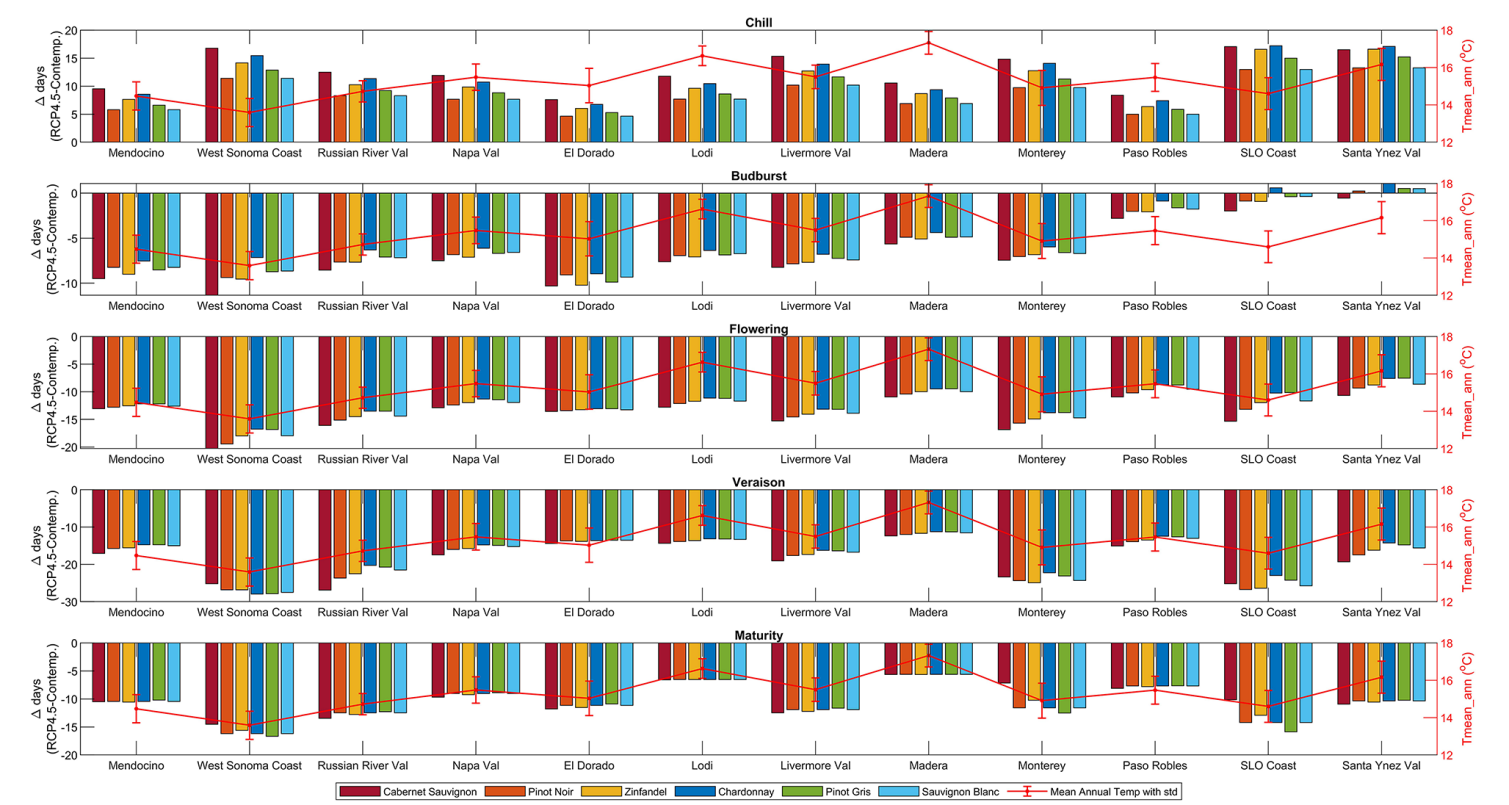
Variety-specific phenological response to climate change across six varieties and 12 AVAs. The left y-axis illustrates the change in timing between the RCP4.5 and the contemporary period for five significant phenological stages (Chill, Budburst, Flowering, Veraison, and Maturity). The 12 American Viticultural Areas (AVAs) are shown along the x-axis in geographical order from north to south. The red lines correspond to the right y-axis and indicate the annual mean temperature during the contemporary period for each AVA, with error bars signifying the standard deviation

When exploring changes in phenology by variety (Fig. 2; Table 4a, Supplemental Fig. 1), which accounts for the phenology model output by variety across the 12 AVAs, those varieties with the lowest chilling degree days (DD_C_) requirement (see Table 2) complete chill accumulation earliest while the highest chill variety considered here, Cabernet Sauvignon, completes chill roughly one month later. Under future conditions, the 12 AVA average chill completion is delayed by approximately 9–13 days across the six varieties, with Cabernet Sauvignon showing the greatest delay. Conversely, the 12 AVA average timing for budburst, flowering, veraison, and maturity advances by an average of 5–7 days, 12–14 days, 17–19 days, and 10–11 days, respectively. At budburst, Cabernet Sauvignon shows the greatest advancement of the red varieties and Pinot Gris of the white varieties. From flowering to maturity the differences between varieties is minimal. All varieties show average advancements in maturity of 10 or 11 days. While the advancement of phenology increases between budburst and veraison, the break in this pattern at maturity is likely attributed to the use of BEDD for determining the timing of maturation as opposed to the accumulation of GDD used for budburst, flowering, veraison stages.

**Table 4a:** The 12-AVA average DOY of chill completion and the onset of four key growing stages (budbreak, flowering, veraison, and maturity) for six grape varieties under contemporary (1991–2020, observed) and future (2040–2069, RCP 4.5) climate conditions. The DOY is computed as the mean of means across 12 California AVAs

Variety	CHILL (DOY)		BUDBREAK (DOY)		FLOWERING (DOY)		VERAISON (DOY)		MATURITY (DOY)	
	1991–2020 Obs.	2040–2069 RCP4.5	1991–2020 Obs.	2040–2069 RCP4.5	1991–2020 Obs.	2040–2069 RCP4.5	1991–2020 Obs.	2040–2069 RCP4.5	1991–2020 Obs.	2040–2069 RCP4.5
**Cabernet Sauvignon**	Dec. 27	Jan. 09	Mar. 28	Mar. 21	May 22	May 08	Aug. 10	Jul. 22	Sep. 23	Sep. 13
**Pinot Noir**	Dec. 01	Dec. 10	Mar. 22	Mar. 16	May 16	May 03	Aug. 02	Jul. 15	Sep. 07	Aug. 28
**Zinfandel**	Dec. 14	Dec. 25	Mar. 28	Mar. 22	May 20	May 08	Aug. 03	Jul. 16	Sep. 13	Sep. 02
**Chardonnay**	Dec. 21	Jan. 02	Mar. 22	Mar. 17	May 19	May 07	Jul. 31	Jul. 14	Sep. 07	Aug. 28
**Pinot Gris**	Dec. 08	Dec. 18	Mar. 26	Mar. 20	May 17	May 05	Jul. 30	Jul. 13	Sep. 02	Aug. 22
**Sauvignon Blanc**	Dec. 01	Dec. 10	Mar. 23	Mar. 18	May 20	May 07	Jul. 31	Jul. 13	Sep. 07	Aug. 28

**Table 4b:** The 6-variety average DOY of chill completion and the onset of four key growing stages (budbreak, flowering, veraison, and maturity) for 12 California AVAs under contemporary (1991–2020, observed) and future (2040–2069, RCP 4.5) climate conditions. The DOY is computed as the mean of means across varieties

AVA	CHILL (DOY)		BUDBREAK (DOY)		FLOWERING (DOY)		VERAISON (DOY)		MATURITY (DOY)	
	1991–2020 Obs.	2040–2069 RCP4.5	1991–2020 Obs.	2040–2069 RCP4.5	1991–2020 Obs.	2040–2069 RCP4.5	1991–2020 Obs.	2040–2069 RCP4.5	1991–2020 Obs.	2040–2069 RCP4.5
**El Dorado**	Dec. 05	Dec. 10	Apr. 04	Mar. 26	May 27	May 14	Jul. 24	Jul. 10	Sep. 12	Aug. 31
**Livermore Valley**	Dec. 15	Dec. 27	Mar. 24	Mar. 17	May 19	May 05	Jul. 29	Jul. 12	Sep. 11	Aug. 30
**Lodi**	Dec. 12	Dec. 22	Mar. 21	Mar. 14	May 07	Apr. 25	Jul. 07	Jun. 23	Aug. 26	Aug. 19
**Madera**	Dec. 11	Dec. 19	Mar. 20	Mar. 15	May 03	Apr. 23	Jun. 29	Jun. 18	Aug. 23	Aug. 17
**Mendocino**	Dec. 04	Dec. 12	Apr. 03	Mar. 25	May 30	May 18	Aug. 06	Jul. 21	Sep. 15	Sep. 05
**Monterey**	Dec. 14	Dec. 26	Mar. 22	Mar. 15	May 19	May 04	Aug. 18	Jul. 25	Sep. 17	Sep. 06
**Napa Valley**	Dec. 11	Dec. 20	Mar. 26	Mar. 19	May 17	May 05	Jul. 24	Jul. 08	Sep. 05	Aug. 27
**Paso Robles**	Dec. 07	Dec. 14	Mar. 24	Mar. 22	May 17	May 08	Jul. 24	Jul. 10	Sep. 01	Aug. 25
**Russian River Valley**	Dec. 10	Dec. 20	Mar. 25	Mar. 18	May 21	May 06	Aug. 10	Jul. 18	Sep. 09	Aug. 27
**Santa Ynez Valley**	Dec. 22	Jan. 06	Mar. 17	Mar. 17	May 09	Apr. 30	Jul. 31	Jul. 14	Sep. 06	Aug. 27
**SLO Coast**	Dec. 22	Jan. 06	Mar. 20	Mar. 20	May 23	May 11	Sep. 04	Aug. 10	Sep. 27	Sep. 13
**West Sonoma Coast**	Dec. 11	Dec. 24	Mar. 30	Mar. 20	Jun. 06	May 18	Sep. 09	Aug. 13	Sep. 29	Sep. 13

### Changes in phenology by AVA

AVA-scale phenology analysis averages the phenology model output across the six varieties for each AVA, providing a view of how general winegrape phenology may shift under climate change at the AVA scale (Table 4b). Through this lens, results show that future phenology timing shifts towards a delay in chill accumulation and an advance in budburst, flowering, veraison and maturation across all AVAs. AVA chill completion is delayed by an average of 5–15 days, with SLO Coast and Santa Ynez Valley AVAs, which have the lowest winter DD_c_ under contemporary conditions, showing the greatest delay. Budburst advances by a week or more in 8 of the 12 AVAs analyzed, with only Madera, Paso Robles, SLO Coast, and Santa Ynez Valley AVAs showing less than seven days of advancement at this stage. Flowering advancements are two weeks or greater in 4 of 12 AVAs (West Sonoma Coast, Livermore Valley, Monterey, Russian River Valley), and four of the AVAs (West Sonoma Coast, Monterey, Russian River Valley, SLO Coast) show veraison advancements of three weeks or greater. Advances in maturity range from less than one week (Madera) to more than two weeks (West Sonoma Coast), with maturity occurring an average of 11 days earlier.

### Geographic influences on phenology

At the AVA-scale, latitude is an important driver of phenology in earlier development phases (i.e., chill completion and budburst), illustrated by generally earlier chill accumulation and later budburst at more northerly AVAs (Fig. 2, Table 4b, Supplemental Table 1). In later phenology phases, climate drivers other than latitude (e.g., ocean proximity, local topography) influence temperature and therefore phenology. For example, moving from flowering to maturity, we observe a subtle contrast between inland and coastal regions with AVAs situated inland, such as El Dorado, Lodi, and Madera, exhibiting a smaller shift in timing compared to coastal AVAs during these later development stages. While these patterns are somewhat muted under future climate conditions as compared to the contemporary, they show one aspect of the influence of geography on phenology. Elevation can also influence temperatures and therefore phenology. At the AVA-scale, results show a positive correlation between elevation and the timing of budburst, suggesting that locations at higher elevations tend to experience a later budburst day; however, this relationship was not significant across all phenology stages (Fig. 2, Supplemental Table 2). Finally, although we focus on the AVA-scale, we note that local topography can also influence phenology. Consider our complementary analysis of projected changes in phenology across two AVAs at comparable latitudes: the topographically complex Napa Valley AVA and the more homogeneous Lodi AVA (Supplemental Fig. 1). There is greater spatial variation in phenology timing over Napa, where across-AVA variations in phenology shifts can be as large as 31 days, as compared to Lodi where phenology shifts are more uniform across stages and varieties, further highlighting the importance of AVA geography on winegrape phenology.

### Changes in agroclimatic metrics

**Fig. 3:**
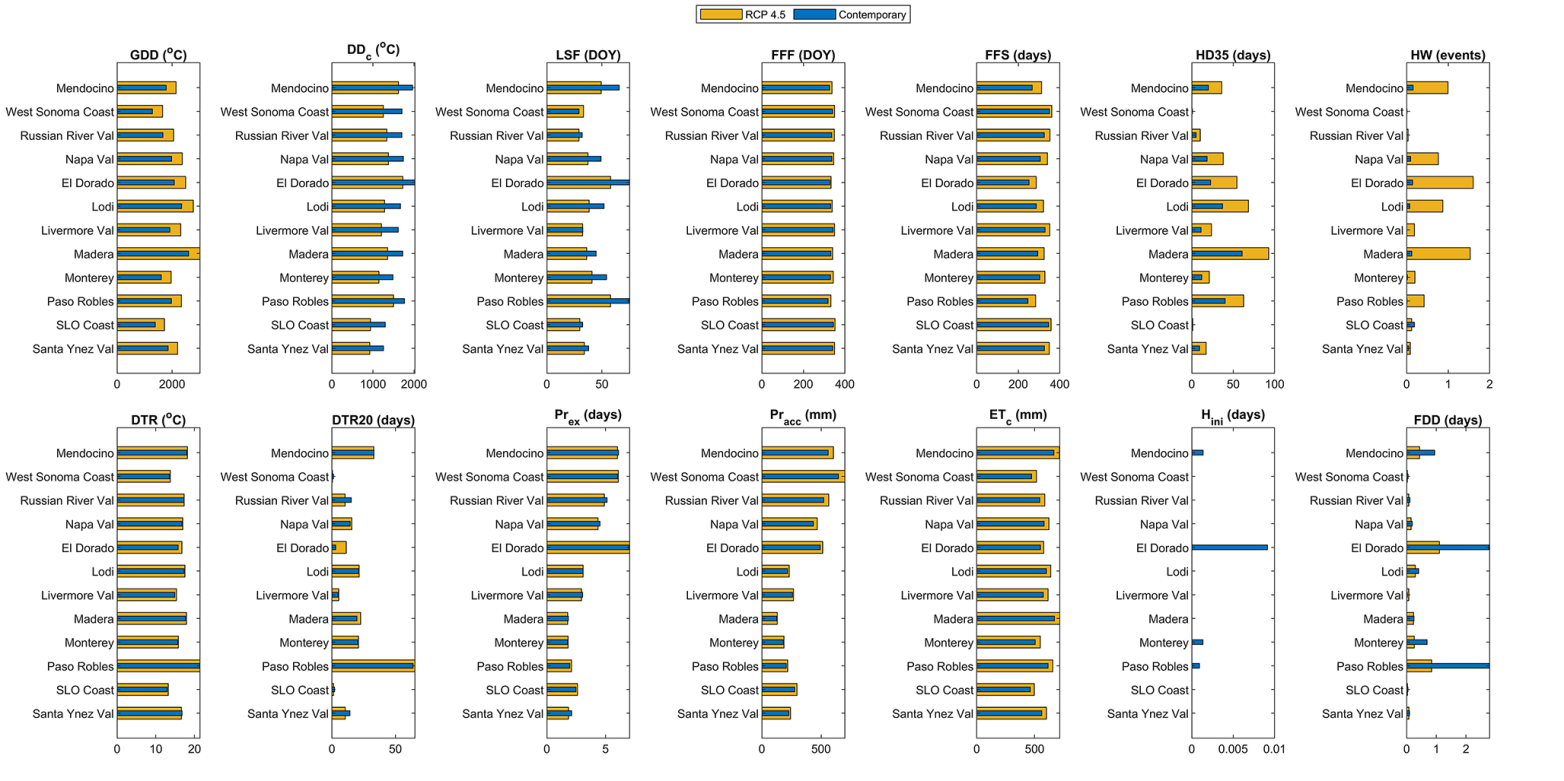
The mean value of 14 general agroclimatic metrics over 12 AVAs for the contemporary period (blue bar) and the future (2040–2069) period under RCP 4.5 (yellow bar). The 12 AVAs are arranged from north to south. The full name of each metric is listed here: Growing Degree Days (GDD), Cold Hardiness (H_ini_), Chilling Degree Days (DD_c_), Frost Damage Days (FDD), Last Spring Freeze (LSF), First Fall Freeze (FFF), Freeze-Free Season (FFS), Hot Days (HD), Heatwaves (HW), Diurnal Temperature Range (DTR), Diurnal Temperature Range > 20 °C (DTR20), Excess Precipitation Days (Pr_ex_), Winter Accumulated Precipitation (Pr_acc_), Crop Evapotranspiration (ET_c_).

Analysis of agroclimatic metrics across the 12 AVAs of interest reveal some notable changes between current and future climates (Fig. 3, Supplemental Tables 2 and 3). Under current climate conditions, expected geographic patterns appear across a number of agroclimatic metrics. For example, inland AVAs (e.g., El Dorado, Lodi, and Madera) exhibit higher Growing Degree Days (GDD) and a greater number of Hot Days above 35 °C (HD35) when compared to their coastal counterparts. Furthermore, metrics like Chilling Degree Days (DD_c_), excess precipitation days (Pr_ex_), and winter accumulated precipitation (Pr_acc_) exhibit a north-to-south gradient, with the northernmost AVA (Mendocino) and the highest elevation region (El Dorado) experiencing the greatest amount of winter chill and excess precipitation days. We also note that higher elevations correlate with increased exposure to days below the threshold for cold hardiness (H_ini_) (Supplemental Table 2).

In regions with greater relative frost risk, such as El Dorado, Mendocino, and Paso Robles, the number of frost damage days (FFD) is projected to decline by approximately 1 to 2 days between the contemporary and future periods. Additionally, we anticipate higher GDD, HD35, and Evapotranspiration (ET_c_) in warmer locations, while lower values of Pr_ex_ and Pr_acc_ are expected in these areas. Analysis shows a positive correlation between annual mean temperature and the change in HD35 (△HD35), suggesting that warmer places are anticipated to experience a higher increase in the number of hot days. Moreover, elevation exhibits a positive correlation with the change in Diurnal Temperature Range (△DTR) and △DTR20, indicating that areas with higher elevations are expected to experience a more pronounced increase in Diurnal Temperature Range and a greater reduction in FDD (Supplemental Table 2). Finally, we note that as with projected phenology changes, projected changes in agroclimatic metrics can be influenced by complex topography within AVAs (Supplemental Fig. 2).

## Discussion

This study offers an analysis that reinforces the existing body of literature on climate effects on agricultural production. Our results corroborate other studies showing that agriculture in California will face the effects of warmer winters and subsequent reduced chill accumulation, longer frost-free seasons, increased evapotranspiration, and more heat extremes (e.g., Cayan et al. 2008; Gershunov and Guirguis 2012; Luedeling et al. 2009; Pathak et al. 2018). Moreover, our results are in line with recent observations of growing season shifts in Napa Valley vineyards (Cayan et al. 2023). Climate extremes have been associated with notable damages to California agriculture (Lobell et al. 2011), and our results show increased exposure to extreme heat under future climate. Heat extremes are a known problem for winegrape cultivation, decreasing berry size and influencing berry chemistry (Greer and Weston 2010; Parker et al. 2020a). In 2021 alone heat was cited as the cause of loss for more than $25 M in crop indemnity claims in Napa and Sonoma counties, two of California’s top wine-producing counties (AgRisk Viewer; Reyes and Elias 2019). Conversely, while other California crops may have to contend with increasingly warm winter temperatures in the form of lower chill accumulation (Luedeling et al. 2009), our results show that due to the low chilling requirements of winegrapes, warmer winters will not see similarly direct negative effects on winegrape cultivation. However, warmer winters – along with reduced frost exposure and longer growing seasons – have the potential to increase pest and disease pressure (Gross, 2021; Pathak et al. 2018).

Just as with non-cultivated plants (e.g., Gordo and Sanz 2010; Polgar and Primack 2011), shifts in crop phenology under climate change have been consistently reported in literature (e.g., Pathak and Stoddard 2018; Pope et al. 2013). In winegrapes, prior studies have shown that future warming will result in earlier development, but the degree of change varies by phenology phase, location, and variety (e.g., Ausseil et al. 2021; Fraga et al. 2016; Webb et al. 2007). While our results align with these studies broadly, there are some distinctions. For example, Fraga et al. (2016) showed that across Europe Pinot Noir harvest timing showed the greatest advancement under projected mid-century conditions, while the changes in timing of flowering were more modest. In contrast, our results show flowering and veraison to have greater advancements, though this is likely due to our use of BEDD to define maturity as compared to the use of a model-simulated estimation of alcohol content by Fraga et al. (2016). Similarly, Webb et al. (2007), investigating climate influence on the phenology of Cabernet Sauvignon and Chardonnay in Australia, suggested greater advancements in maturity than our results show. Although these distinctions highlight the role of modeling approach in understanding phenology changes, our overall findings align with these prior studies and add to the body of literature underscoring the importance of geography and variety in climate-driven phenological shifts.

Although these results capture the overarching effects of climate change on winegrape production in California, there are some limitations to our study. The thresholds used in our phenology models come from trials conducted under different environmental conditions and outside of California and as such may not be precise relative to the thresholds (general or variety specific) that may emerge were these trials replicated in our AVAs of interest. For some metrics, there is a lack of variety-specific threshold data in the literature which limits our results. For example, while it has been suggested that cooler-climate winegrape varieties benefit from larger DTR than warmer-climate varieties (Jones 2015), variety-specific optimal DTR for our varieties is lacking. For metrics like BEDD, the limitations lie in their design; BEDD is not designed to model maturation as defined by a specific level of ripening (e.g., 24 °Brix). BEDD may limit the ability to capture warming-driven shifts in the timing of generalized maturity due to the T_upper_ placing a cap on heat accumulation, suggesting that BEDD may not be ideally suited for climate change modeling applications (Hall and Jones 2010). Even using an ideal metric for modeling thermally-driven development may still produce imprecise magnitudes of phenological change (e.g. Sadras and Moran 2013; Wolkovich et al. 2012). Beyond these thresholds and metrics limitations, it is important to acknowledge the spatial limitations of the climate data relative to the microclimatic factors that can influence winegrape phenology and alter climate exposure at the vineyard scale. It is also critical to acknowledge that these results do not account for the myriad farm management actions that growers can implement to mitigate exposure to or impacts of undesirable conditions.

The adoption of climate-smart adaptation practices can improve growers’ resilience to climate change and help ameliorate the negative impacts of a warming world. For California winegrape growers faced with greater water demands by way of higher crop evapotranspiration and a need to alleviate the impacts of increasing exposure to heat extremes, climate-smart adaptation practices may include improving soil structure and incorporating soil amendments to increase soil water holding capacity; improving irrigation management to increase infiltration and improve water use efficiency; adopting minimum tillage or cover cropping practices to reduce soil water evaporation; planting new drought- and heat-tolerant rootstocks and varieties; and managing the vineyard for heat exposure through canopy-management practices or the installation of heat-reducing shade netting (Parker et al. 2023). Providing information at meaningful scales has been identified as a key component to encouraging the adoption of adaptation practices (Johnson et al. 2023). In earlier work, Babin et al. (2022) showed that the presentation of climate change projections at the local scale to vineyard managers and technical service providers (TSPs) promotes the consideration of adaptation strategies in vineyard management planning. Here we address both AVA-scale climate projections and projected phenology, providing variety-specific information at a meaningful spatial scale that can empower growers to identify and adopt the most appropriate adaptation actions for their situation.

## Conclusions

Through quantifying these projected changes in phenology and agroclimatic metrics at the variety-specific and AVA scale, we offer information at a resolution that can support grower and industry decision-making. While the methodological approach employed here can be applied to other varieties and regions within and beyond California’s borders, we recommend continued field trials to not only ensure accurate variety-specific bioclimate information, but also to attempt to elucidate the complex relationships between climate, variety, and other aspects of the vine (e.g., rootstock), the vineyard system (e.g., soils), and adaptive water and nutrient management practices. Ultimately, model outputs are only as good as their inputs and better understanding of these complex relationships will be needed to improve modeling for decision support and the long-term resilience of viticulture under climate change.

### Electronic supplementary material

Below is the link to the electronic supplementary material.

Supplementary Material 1

Supplementary Material 2

Supplementary Material 3
