# Supplementary figures and images for: A variety-specific analysis of climate change effects on California winegrapes
Source: Int J Biometeorol. 2024 Apr 23;68(8):1559–71. doi: 10.1007/s00484-024-02684-8 (PMC11282142; doi:10.1007/s00484-024-02684-8)

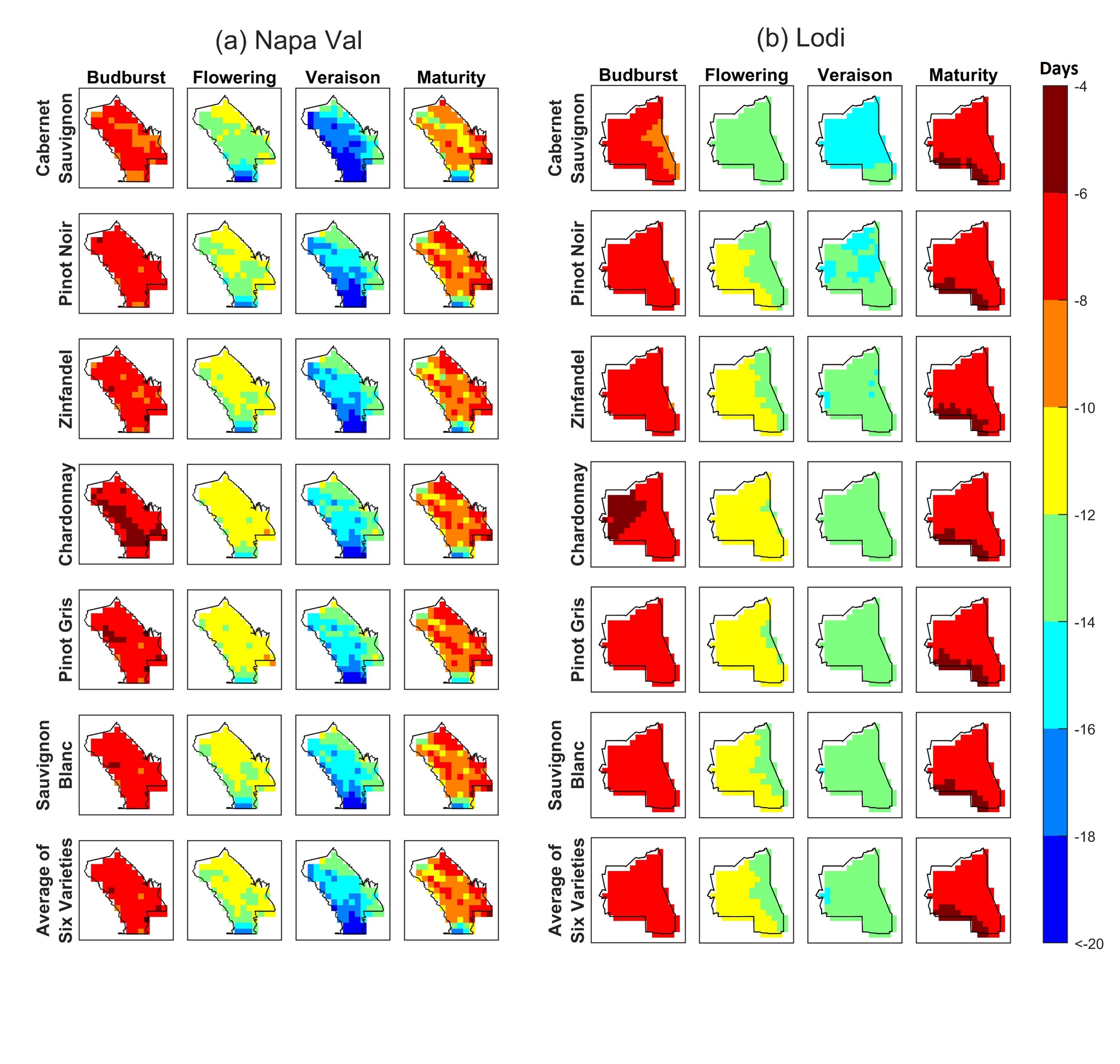

Supplement: Supplementary file 1 — Supplementary Material 1 [file 484_2024_2684_MOESM1_ESM.jpg]

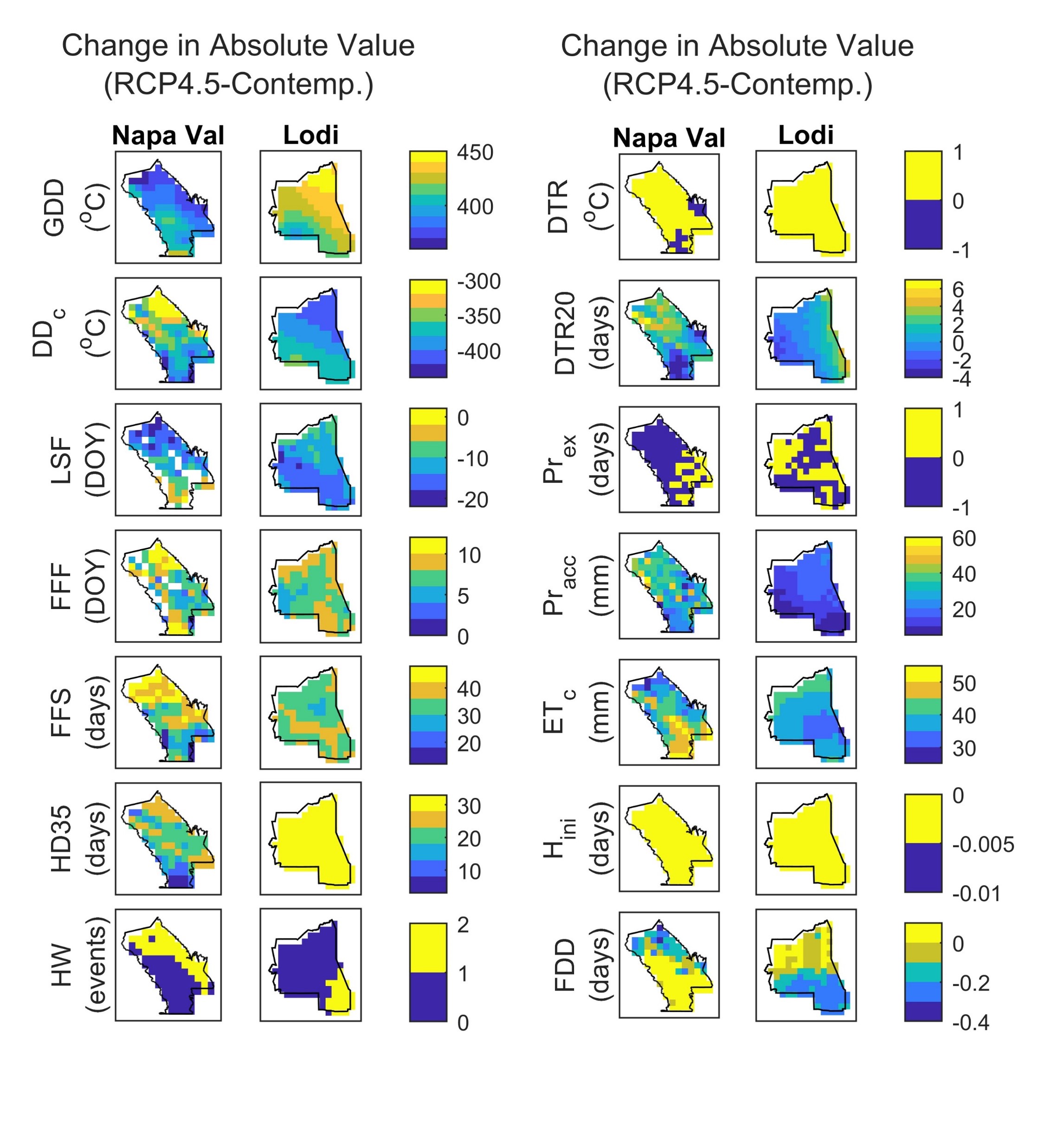

Supplement: Supplementary file 2 — Supplementary Material 2 [file 484_2024_2684_MOESM2_ESM.jpg]
