# Supplementary material for: A variety-specific analysis of climate change effects on California winegrapes
Source: Int J Biometeorol. 2024 Apr 23;68(8):1559–71. doi: 10.1007/s00484-024-02684-8 (PMC11282142; doi:10.1007/s00484-024-02684-8)
Supplement: Supplementary file 3 — Supplementary Material 3 [file 484_2024_2684_MOESM3_ESM.docx]

**Supplemental Material**

**Supplemental Table 1.** The timing (day of the year, DOY) of chill completion and four key growing stages (budbreak, flowering, veraison, and maturity) for six grape varieties across 12 California AVAs under contemporary (1991-2020, observed) and future (2040-2069, RCP 4.5) climate conditions. The trend for each stage over each time period is also reported, with statistically significant trends in bold. Note: For DOY greater than 365, this indicates occurrence in the following year. To determine the calendar DOY, subtract 365 from the given number.

| **Mendocino** |  | **Chill Completion (DOY)** | | **BUDBREAK (DOY)** | | **BLOOM (DOY)** | | **VERAISON (DOY)** | | **MATURITY (DOY)** | |
| --- | --- | --- | --- | --- | --- | --- | --- | --- | --- | --- | --- |
|  |  | **1991-2020, obs.** | **2040-2069, RCP4.5** | **1991-2020, obs.** | **2040-2069, RCP4.5** | **1991-2020, obs.** | **2040-2069, RCP4.5** | **1991-2020, obs.** | **2040-2069, RCP4.5** | **1991-2020, obs.** | **2040-2069, RCP4.5** |
| **Cabernet Sauvignon** | Mean | 352 | 361 | 97 | 88 | 153 | 140 | 224 | 207 | 272 | 262 |
|  | (Min, Max) | (336, 379) | (348, 379) | (73, 136) | (78, 106) | (120, 192) | (123, 157) | (194, 310) | (191, 244) | (250, 304) | (251, 282) |
|  | Trend | 0.18 | **0.27** | -0.05 | **-0.23** | -0.46 | **-0.24** | -0.53 | **-0.24** | -0.3 | **-0.17** |
| **Pinot Noir** | Mean | 329 | 334 | 90 | 82 | 148 | 135 | 217 | 201 | 255 | 245 |
|  | (Min, Max) | (319, 349) | (325, 346) | (71, 126) | (74, 98) | (117, 185) | (119, 153) | (187, 304) | (186, 233) | (233, 302) | (234, 264) |
|  | Trend | 0.09 | **0.22** | 0.05 | **-0.18** | -0.51 | **-0.24** | -0.43 | **-0.23** | -0.33 | **-0.16** |
| **Zinfandel** | Mean | 341 | 348 | 97 | 88 | 151 | 139 | 218 | 203 | 261 | 250 |
|  | (Min, Max) | (327, 364) | (337, 363) | (75, 131) | (79, 105) | (122, 185) | (123, 156) | (190, 292) | (189, 233) | (239, 302) | (240, 271) |
|  | Trend | 0.13 | **0.23** | 0.03 | **-0.21** | -0.39 | **-0.23** | -0.42 | **-0.23** | -0.33 | **-0.16** |
| **Chardonnay** | Mean | 346 | 355 | 89 | 81 | 150 | 137 | 216 | 201 | 255 | 245 |
|  | (Min, Max) | (331, 370) | (343, 373) | (72, 122) | (75, 95) | (121, 182) | (123, 155) | (189, 274) | (188, 228) | (233, 302) | (234, 264) |
|  | Trend | 0.16 | **0.24** | 0.1 | **-0.15** | -0.41 | **-0.22** | -0.37 | **-0.22** | -0.33 | **-0.16** |
| **Pinot Gris** | Mean | 335 | 341 | 94 | 86 | 148 | 136 | 215 | 200 | 249 | 239 |
|  | (Min, Max) | (323, 359) | (331, 354) | (74, 127) | (78, 102) | (120, 179) | (121, 153) | (188, 276) | (186, 228) | (228, 299) | (229, 258) |
|  | Trend | 0.09 | **0.23** | 0.03 | **-0.2** | -0.43 | **-0.22** | -0.39 | **-0.22** | -0.32 | **-0.16** |
| **Sauvignon Blanc** | Mean | 329 | 334 | 91 | 83 | 151 | 139 | 215 | 200 | 255 | 245 |
|  | (Min, Max) | (319, 349) | (325, 346) | (72, 126) | (75, 98) | (121, 185) | (123, 156) | (187, 286) | (185, 229) | (233, 302) | (234, 264) |
|  | Trend | 0.09 | **0.22** | 0.06 | **-0.18** | -0.41 | **-0.23** | -0.41 | **-0.23** | -0.33 | **-0.16** |
|  |  |  | |  | |  | |  | |  | |
| **West Sonoma Coast** |  | **Chill Completion (DOY)** | | **BUDBREAK (DOY)** | | **BLOOM (DOY)** | | **VERAISON (DOY)** | | **MATURITY (DOY)** | |
|  |  | **1991-2020, obs.** | **2040-2069, RCP4.5** | **1991-2020, obs.** | **2040-2069, RCP4.5** | **1991-2020, obs.** | **2040-2069, RCP4.5** | **1991-2020, obs.** | **2040-2069, RCP4.5** | **1991-2020, obs.** | **2040-2069, RCP4.5** |
| **Cabernet Sauvignon** | Mean | 359 | 376 | 93 | 82 | 163 | 143 | 263 | 238 | 284 | 270 |
|  | (Min, Max) | (341, 397) | (364, 387) | (74, 136) | (76, 90) | (129, 231) | (126, 173) | (226, 328) | (210, 313) | (255, 304) | (253, 302) |
|  | Trend | 0.18 | **0.32** | 0.16 | **-0.15** | -0.16 | **-0.26** | -0.18 | **-0.4** | 0.13 | **-0.19** |
| **Pinot Noir** | Mean | 334 | 346 | 86 | 76 | 155 | 136 | 254 | 227 | 270 | 254 |
|  | (Min, Max) | (321, 358) | (336, 355) | (71, 125) | (72, 82) | (122, 217) | (121, 162) | (213, 335) | (202, 298) | (239, 304) | (237, 304) |
|  | Trend | 0.08 | **0.27** | 0.16 | **-0.11** | -0.08 | **-0.27** | -0.1 | **-0.38** | 0.15 | **-0.24** |
| **Zinfandel** | Mean | 347 | 361 | 92 | 83 | 158 | 140 | 253 | 226 | 275 | 259 |
|  | (Min, Max) | (331, 371) | (351, 377) | (76, 124) | (78, 89) | (128, 212) | (126, 164) | (214, 339) | (203, 292) | (244, 304) | (242, 301) |
|  | Trend | 0.16 | **0.32** | 0.16 | **-0.13** | -0.05 | **-0.23** | -0.1 | **-0.35** | 0.17 | **-0.21** |
| **Chardonnay** | Mean | 353 | 369 | 84 | 77 | 154 | 137 | 248 | 220 | 270 | 254 |
|  | (Min, Max) | (337, 384) | (357, 380) | (73, 111) | (73, 81) | (126, 204) | (125, 158) | (211, 340) | (200, 273) | (239, 304) | (237, 304) |
|  | Trend | 0.17 | **0.34** | 0.18 | **-0.08** | 0.01 | **-0.22** | -0.05 | **-0.31** | 0.15 | **-0.24** |
| **Pinot Gris** | Mean | 341 | 354 | 90 | 81 | 152 | 135 | 248 | 220 | 265 | 248 |
|  | (Min, Max) | (326, 364) | (344, 367) | (75, 119) | (76, 86) | (123, 201) | (122, 156) | (210, 344) | (199, 278) | (233, 304) | (231, 297) |
|  | Trend | 0.12 | **0.3** | 0.21 | **-0.11** | 0.01 | **-0.22** | -0.11 | **-0.32** | 0.17 | **-0.23** |
| **Sauvignon Blanc** | Mean | 334 | 346 | 87 | 78 | 157 | 139 | 249 | 221 | 270 | 254 |
|  | (Min, Max) | (321, 358) | (336, 355) | (73, 118) | (74, 83) | (127, 212) | (125, 163) | (211, 341) | (199, 287) | (239, 304) | (237, 304) |
|  | Trend | 0.08 | **0.27** | 0.19 | **-0.1** | -0.02 | **-0.24** | -0.17 | **-0.34** | 0.15 | **-0.24** |
|  |  |  |  |  |  |  |  |  |  |  |  |
| **Russian River Valley** |  | **Chill Completion (DOY)** | | **BUDBREAK (DOY)** | | **BLOOM (DOY)** | | **VERAISON (DOY)** | | **MATURITY (DOY)** | |
|  |  | **1991-2020, obs.** | **2040-2069, RCP4.5** | **1991-2020, obs.** | **2040-2069, RCP4.5** | **1991-2020, obs.** | **2040-2069, RCP4.5** | **1991-2020, obs.** | **2040-2069, RCP4.5** | **1991-2020, obs.** | **2040-2069, RCP4.5** |
| **Cabernet Sauvignon** | Mean | 359 | 371 | 87 | 78 | 144 | 128 | 233 | 206 | 267 | 253 |
|  | (Min, Max) | (342, 386) | (360, 385) | (73, 116) | (74, 83) | (117, 169) | (115, 139) | (193, 282) | (186, 225) | (245, 301) | (245, 265) |
|  | Trend | 0.12 | **0.31** | 0.18 | **-0.13** | -0.16 | **-0.26** | -0.15 | **-0.31** | 0.02 | **-0.15** |
| **Pinot Noir** | Mean | 333 | 342 | 81 | 74 | 138 | 123 | 222 | 198 | 249 | 236 |
|  | (Min, Max) | (322, 359) | (334, 352) | (70, 110) | (70, 78) | (115, 164) | (111, 133) | (187, 268) | (180, 215) | (229, 280) | (229, 248) |
|  | Trend | -0.01 | **0.25** | 0.1 | **-0.1** | -0.2 | **-0.25** | -0.06 | **-0.29** | 0.04 | **-0.14** |
| **Zinfandel** | Mean | 347 | 357 | 87 | 79 | 142 | 127 | 222 | 200 | 255 | 242 |
|  | (Min, Max) | (332, 370) | (348, 372) | (74, 113) | (76, 83) | (117, 165) | (116, 136) | (188, 264) | (182, 216) | (234, 286) | (234, 253) |
|  | Trend | 0.08 | **0.27** | 0.16 | **-0.12** | -0.1 | **-0.23** | -0.08 | **-0.28** | 0.04 | **-0.14** |
| **Chardonnay** | Mean | 353 | 364 | 81 | 75 | 140 | 126 | 218 | 197 | 249 | 236 |
|  | (Min, Max) | (338, 381) | (354, 380) | (72, 103) | (72, 78) | (117, 162) | (116, 134) | (188, 253) | (181, 212) | (229, 280) | (229, 248) |
|  | Trend | 0.09 | **0.29** | 0.09 | **-0.08** | -0.09 | **-0.21** | -0.05 | **-0.26** | 0.04 | **-0.14** |
| **Pinot Gris** | Mean | 340 | 349 | 85 | 78 | 138 | 124 | 217 | 196 | 243 | 231 |
|  | (Min, Max) | (327, 365) | (341, 362) | (74, 110) | (75, 82) | (116, 160) | (114, 133) | (186, 254) | (180, 211) | (223, 273) | (223, 242) |
|  | Trend | 0.07 | **0.25** | 0.16 | **-0.1** | -0.11 | **-0.21** | -0.06 | **-0.26** | 0.04 | **-0.14** |
| **Sauvignon Blanc** | Mean | 333 | 342 | 82 | 75 | 142 | 127 | 218 | 196 | 249 | 236 |
|  | (Min, Max) | (322, 359) | (334, 352) | (72, 109) | (72, 79) | (117, 165) | (116, 136) | (186, 258) | (179, 212) | (229, 280) | (229, 248) |
|  | Trend | -0.01 | **0.25** | 0.12 | **-0.1** | -0.07 | **-0.23** | -0.07 | **-0.27** | 0.04 | **-0.14** |
|  |  |  |  |  |  |  |  |  |  |  |  |
| **Napa Valley** |  | **Chill Completion (DOY)** | | **BUDBREAK (DOY)** | | **BLOOM (DOY)** | | **VERAISON (DOY)** | | **MATURITY (DOY)** | |
|  |  | **1991-2020, obs.** | **2040-2069, RCP4.5** | **1991-2020, obs.** | **2040-2069, RCP4.5** | **1991-2020, obs.** | **2040-2069, RCP4.5** | **1991-2020, obs.** | **2040-2069, RCP4.5** | **1991-2020, obs.** | **2040-2069, RCP4.5** |
| **Cabernet Sauvignon** | Mean | 359 | 371 | 88 | 80 | 139 | 127 | 211 | 194 | 263 | 253 |
|  | (Min, Max) | (338, 391) | (355, 389) | (72, 120) | (72, 94) | (115, 175) | (112, 146) | (177, 274) | (175, 213) | (242, 303) | (243, 272) |
|  | Trend | 0.13 | **0.29** | 0.12 | **-0.15** | -0.33 | **-0.23** | -0.2 | **-0.26** | -0.22 | **-0.14** |
| **Pinot Noir** | Mean | 334 | 342 | 82 | 75 | 135 | 122 | 204 | 188 | 245 | 236 |
|  | (Min, Max) | (319, 359) | (332, 356) | (70, 118) | (67, 87) | (111, 172) | (108, 141) | (172, 261) | (170, 207) | (225, 290) | (227, 253) |
|  | Trend | 0.06 | **0.25** | 0.05 | **-0.12** | -0.24 | **-0.23** | -0.15 | **-0.25** | -0.18 | **-0.14** |
| **Zinfandel** | Mean | 347 | 357 | 88 | 81 | 139 | 127 | 206 | 190 | 251 | 242 |
|  | (Min, Max) | (329, 381) | (344, 377) | (74, 120) | (74, 94) | (115, 173) | (113, 145) | (176, 258) | (173, 208) | (231, 299) | (232, 259) |
|  | Trend | 0.1 | **0.27** | 0.09 | **-0.14** | -0.25 | **-0.21** | -0.15 | **-0.24** | -0.19 | **-0.14** |
| **Chardonnay** | Mean | 353 | 364 | 82 | 76 | 137 | 126 | 204 | 189 | 245 | 236 |
|  | (Min, Max) | (333, 386) | (349, 383) | (71, 116) | (69, 85) | (115, 172) | (113, 143) | (175, 249) | (173, 207) | (225, 290) | (227, 253) |
|  | Trend | 0.13 | **0.28** | 0.05 | **-0.1** | -0.17 | **-0.2** | -0.13 | **-0.23** | -0.18 | **-0.14** |
| **Pinot Gris** | Mean | 341 | 350 | 86 | 80 | 135 | 124 | 203 | 188 | 239 | 230 |
|  | (Min, Max) | (324, 371) | (338, 366) | (73, 119) | (73, 91) | (114, 171) | (111, 141) | (174, 249) | (172, 206) | (220, 280) | (221, 248) |
|  | Trend | 0.1 | **0.26** | 0.11 | **-0.12** | -0.21 | **-0.21** | -0.14 | **-0.23** | -0.17 | **-0.14** |
| **Sauvignon Blanc** | Mean | 334 | 342 | 83 | 77 | 138 | 127 | 202 | 187 | 245 | 236 |
|  | (Min, Max) | (319, 359) | (332, 356) | (71, 118) | (69, 88) | (115, 173) | (113, 145) | (172, 251) | (171, 206) | (225, 290) | (227, 253) |
|  | Trend | 0.06 | **0.25** | 0.06 | **-0.12** | -0.18 | **-0.2** | -0.16 | **-0.24** | -0.18 | **-0.14** |
|  |  |  |  |  |  |  |  |  |  |  |  |
| **El Dorado** |  | **Chill Completion (DOY)** | | **BUDBREAK (DOY)** | | **BLOOM (DOY)** | | **VERAISON (DOY)** | | **MATURITY (DOY)** | |
|  |  | **1991-2020, obs.** | **2040-2069, RCP4.5** | **1991-2020, obs.** | **2040-2069, RCP4.5** | **1991-2020, obs.** | **2040-2069, RCP4.5** | **1991-2020, obs.** | **2040-2069, RCP4.5** | **1991-2020, obs.** | **2040-2069, RCP4.5** |
| **Cabernet Sauvignon** | Mean | 351 | 359 | 98 | 88 | 149 | 135 | 208 | 194 | 269 | 258 |
|  | (Min, Max) | (335, 379) | (345, 376) | (74, 144) | (75, 110) | (117, 186) | (119, 154) | (185, 244) | (180, 212) | (247, 304) | (247, 273) |
|  | Trend | 0.2 | **0.23** | -0.03 | **-0.22** | -0.16 | **-0.23** | -0.18 | **-0.23** | -0.01 | **-0.15** |
| **Pinot Noir** | Mean | 329 | 334 | 91 | 82 | 145 | 131 | 203 | 189 | 252 | 241 |
|  | (Min, Max) | (318, 352) | (325, 345) | (71, 136) | (71, 101) | (115, 182) | (114, 150) | (181, 237) | (175, 207) | (230, 296) | (231, 254) |
|  | Trend | 0.13 | **0.17** | 0.02 | **-0.18** | -0.22 | **-0.23** | -0.2 | **-0.23** | -0.02 | **-0.14** |
| **Zinfandel** | Mean | 341 | 347 | 98 | 88 | 148 | 135 | 205 | 192 | 258 | 246 |
|  | (Min, Max) | (326, 362) | (335, 360) | (76, 142) | (76, 108) | (119, 185) | (119, 154) | (183, 240) | (178, 209) | (235, 300) | (236, 260) |
|  | Trend | 0.13 | **0.2** | -0.01 | **-0.2** | -0.17 | **-0.22** | -0.17 | **-0.22** | -0.02 | **-0.14** |
| **Chardonnay** | Mean | 346 | 353 | 90 | 81 | 147 | 134 | 205 | 191 | 252 | 241 |
|  | (Min, Max) | (331, 370) | (341, 368) | (73, 128) | (72, 98) | (118, 184) | (119, 153) | (183, 239) | (177, 209) | (230, 296) | (231, 254) |
|  | Trend | 0.18 | **0.21** | 0.04 | **-0.15** | -0.14 | **-0.21** | -0.11 | **-0.22** | -0.02 | **-0.14** |
| **Pinot Gris** | Mean | 335 | 340 | 96 | 86 | 146 | 133 | 203 | 190 | 246 | 235 |
|  | (Min, Max) | (322, 356) | (331, 353) | (75, 140) | (75, 105) | (117, 182) | (117, 151) | (182, 238) | (176, 207) | (224, 295) | (225, 248) |
|  | Trend | 0.14 | **0.18** | 0.02 | **-0.19** | -0.17 | **-0.21** | -0.11 | **-0.23** | -0.01 | **-0.14** |
| **Sauvignon Blanc** | Mean | 329 | 334 | 92 | 83 | 149 | 135 | 203 | 189 | 252 | 241 |
|  | (Min, Max) | (318, 352) | (325, 345) | (73, 135) | (72, 101) | (118, 186) | (119, 153) | (180, 237) | (175, 207) | (230, 296) | (231, 254) |
|  | Trend | 0.13 | **0.17** | 0.02 | **-0.18** | -0.14 | **-0.22** | -0.13 | **-0.23** | -0.02 | **-0.14** |
|  |  |  | |  | |  | |  | |  | |
| **Lodi** |  | **Chill Completion (DOY)** | | **BUDBREAK (DOY)** | | **BLOOM (DOY)** | | **VERAISON (DOY)** | | **MATURITY (DOY)** | |
|  |  | **1991-2020, obs.** | **2040-2069, RCP4.5** | **1991-2020, obs.** | **2040-2069, RCP4.5** | **1991-2020, obs.** | **2040-2069, RCP4.5** | **1991-2020, obs.** | **2040-2069, RCP4.5** | **1991-2020, obs.** | **2040-2069, RCP4.5** |
| **Cabernet Sauvignon** | Mean | 360 | 372 | 82 | 74 | 128 | 115 | 192 | 177 | 252 | 245 |
|  | (Min, Max) | (343, 382) | (362, 385) | (73, 103) | (71, 78) | (113, 152) | (109, 124) | (176, 215) | (171, 185) | (242, 273) | (242, 251) |
|  | Trend | 0.16 | **0.27** | 0.07 | **-0.09** | -0.17 | **-0.22** | -0.13 | **-0.23** | -0.07 | **-0.1** |
| **Pinot Noir** | Mean | 336 | 343 | 77 | 70 | 124 | 112 | 186 | 172 | 235 | 228 |
|  | (Min, Max) | (323, 358) | (336, 351) | (70, 96) | (66, 73) | (107, 146) | (106, 120) | (170, 210) | (166, 180) | (225, 255) | (225, 234) |
|  | Trend | 0.09 | **0.22** | 0.01 | **-0.08** | -0.17 | **-0.21** | -0.1 | **-0.22** | -0.07 | **-0.1** |
| **Zinfandel** | Mean | 348 | 358 | 82 | 75 | 128 | 116 | 189 | 175 | 240 | 234 |
|  | (Min, Max) | (334, 370) | (351, 365) | (75, 101) | (72, 78) | (114, 150) | (110, 124) | (173, 210) | (169, 182) | (231, 260) | (231, 240) |
|  | Trend | 0.13 | **0.23** | 0.06 | **-0.08** | -0.16 | **-0.19** | -0.11 | **-0.21** | -0.07 | **-0.1** |
| **Chardonnay** | Mean | 355 | 365 | 78 | 71 | 127 | 116 | 188 | 175 | 235 | 228 |
|  | (Min, Max) | (339, 377) | (357, 374) | (72, 92) | (68, 74) | (114, 148) | (110, 123) | (173, 208) | (169, 182) | (225, 255) | (225, 234) |
|  | Trend | 0.16 | **0.26** | 0.02 | **-0.07** | -0.13 | **-0.18** | -0.05 | **-0.21** | -0.07 | **-0.1** |
| **Pinot Gris** | Mean | 342 | 351 | 81 | 74 | 125 | 114 | 187 | 173 | 229 | 223 |
|  | (Min, Max) | (329, 364) | (344, 359) | (74, 98) | (71, 77) | (112, 145) | (109, 122) | (172, 207) | (168, 180) | (219, 249) | (220, 228) |
|  | Trend | 0.12 | **0.23** | 0.05 | **-0.08** | -0.11 | **-0.18** | -0.08 | **-0.21** | -0.07 | **-0.1** |
| **Sauvignon Blanc** | Mean | 336 | 343 | 78 | 72 | 128 | 116 | 186 | 172 | 235 | 228 |
|  | (Min, Max) | (323, 358) | (336, 351) | (72, 96) | (68, 75) | (114, 150) | (110, 124) | (170, 207) | (167, 179) | (225, 255) | (225, 234) |
|  | Trend | 0.09 | **0.22** | 0.04 | **-0.07** | -0.14 | **-0.19** | -0.08 | **-0.22** | -0.07 | **-0.1** |
|  |  |  |  |  |  |  |  |  |  |  |  |
| **Livermore Valley** |  | **Chill Completion (DOY)** | | **BUDBREAK (DOY)** | | **BLOOM (DOY)** | | **VERAISON (DOY)** | | **MATURITY (DOY)** | |
|  |  | **1991-2020, obs.** | **2040-2069, RCP4.5** | **1991-2020, obs.** | **2040-2069, RCP4.5** | **1991-2020, obs.** | **2040-2069, RCP4.5** | **1991-2020, obs.** | **2040-2069, RCP4.5** | **1991-2020, obs.** | **2040-2069, RCP4.5** |
| **Cabernet Sauvignon** | Mean | 363 | 379 | 86 | 78 | 141 | 126 | 217 | 198 | 268 | 256 |
|  | (Min, Max) | (342, 405) | (365, 398) | (73, 121) | (73, 87) | (118, 176) | (115, 141) | (190, 270) | (186, 217) | (246, 304) | (248, 269) |
|  | Trend | 0.24 | **0.32** | 0.11 | **-0.13** | -0.23 | **-0.23** | -0.19 | **-0.28** | -0.08 | **-0.16** |
| **Pinot Noir** | Mean | 338 | 348 | 81 | 73 | 136 | 121 | 209 | 192 | 251 | 239 |
|  | (Min, Max) | (322, 365) | (339, 365) | (70, 117) | (67, 80) | (113, 169) | (111, 136) | (185, 254) | (180, 210) | (229, 292) | (231, 252) |
|  | Trend | 0.14 | **0.26** | 0.03 | **-0.11** | -0.22 | **-0.24** | -0.18 | **-0.25** | -0.08 | **-0.15** |
| **Zinfandel** | Mean | 351 | 364 | 86 | 79 | 140 | 126 | 211 | 194 | 257 | 244 |
|  | (Min, Max) | (332, 387) | (352, 383) | (75, 119) | (74, 86) | (119, 171) | (116, 139) | (186, 253) | (182, 210) | (235, 304) | (237, 258) |
|  | Trend | 0.18 | **0.28** | 0.09 | **-0.12** | -0.18 | **-0.21** | -0.19 | **-0.25** | -0.1 | **-0.16** |
| **Chardonnay** | Mean | 357 | 371 | 81 | 74 | 138 | 125 | 208 | 192 | 251 | 239 |
|  | (Min, Max) | (338, 391) | (358, 390) | (72, 111) | (69, 80) | (118, 168) | (116, 137) | (186, 245) | (181, 208) | (229, 292) | (231, 252) |
|  | Trend | 0.19 | **0.31** | 0.05 | **-0.09** | -0.16 | **-0.2** | -0.17 | **-0.24** | -0.08 | **-0.15** |
| **Pinot Gris** | Mean | 345 | 356 | 85 | 77 | 136 | 123 | 207 | 191 | 245 | 233 |
|  | (Min, Max) | (327, 381) | (346, 377) | (74, 118) | (73, 84) | (117, 167) | (114, 136) | (184, 245) | (180, 207) | (224, 284) | (226, 246) |
|  | Trend | 0.16 | **0.27** | 0.09 | **-0.11** | -0.2 | **-0.2** | -0.17 | **-0.24** | -0.07 | **-0.15** |
| **Sauvignon Blanc** | Mean | 338 | 348 | 82 | 74 | 140 | 126 | 207 | 191 | 251 | 239 |
|  | (Min, Max) | (322, 365) | (339, 365) | (72, 117) | (69, 81) | (118, 170) | (116, 139) | (183, 247) | (179, 207) | (229, 292) | (231, 252) |
|  | Trend | 0.14 | **0.26** | 0.09 | **-0.11** | -0.18 | **-0.21** | -0.18 | **-0.25** | -0.08 | **-0.15** |
|  |  |  |  |  |  |  |  |  |  |  |  |
| **Madera** |  | **Chill Completion (DOY)** | | **BUDBREAK (DOY)** | | **BLOOM (DOY)** | | **VERAISON (DOY)** | | **MATURITY (DOY)** | |
|  |  | **1991-2020, obs.** | **2040-2069, RCP4.5** | **1991-2020, obs.** | **2040-2069, RCP4.5** | **1991-2020, obs.** | **2040-2069, RCP4.5** | **1991-2020, obs.** | **2040-2069, RCP4.5** | **1991-2020, obs.** | **2040-2069, RCP4.5** |
| **Cabernet Sauvignon** | Mean | 358 | 369 | 80 | 75 | 124 | 113 | 183 | 171 | 249 | 243 |
|  | (Min, Max) | (343, 380) | (359, 381) | (72, 101) | (68, 81) | (107, 150) | (106, 120) | (168, 207) | (166, 177) | (239, 266) | (241, 246) |
|  | Trend | 0.25 | **0.23** | 0.03 | **-0.08** | -0.2 | **-0.17** | -0.15 | **-0.2** | -0.1 | **-0.07** |
| **Pinot Noir** | Mean | 335 | 342 | 76 | 71 | 120 | 109 | 178 | 166 | 232 | 226 |
|  | (Min, Max) | (323, 354) | (335, 349) | (68, 95) | (64, 78) | (103, 144) | (103, 116) | (160, 201) | (161, 172) | (222, 248) | (224, 230) |
|  | Trend | 0.13 | **0.18** | -0.03 | **-0.06** | -0.1 | **-0.17** | -0.16 | **-0.19** | -0.11 | **-0.07** |
| **Zinfandel** | Mean | 347 | 355 | 81 | 76 | 124 | 114 | 181 | 169 | 237 | 232 |
|  | (Min, Max) | (333, 366) | (348, 365) | (74, 99) | (70, 82) | (109, 149) | (108, 121) | (165, 203) | (164, 175) | (228, 254) | (230, 235) |
|  | Trend | 0.18 | **0.2** | 0.04 | **-0.07** | -0.2 | **-0.15** | -0.16 | **-0.19** | -0.1 | **-0.07** |
| **Chardonnay** | Mean | 353 | 362 | 77 | 72 | 124 | 114 | 181 | 170 | 232 | 226 |
|  | (Min, Max) | (339, 372) | (354, 373) | (71, 92) | (66, 79) | (109, 146) | (108, 121) | (166, 202) | (164, 175) | (222, 248) | (224, 230) |
|  | Trend | 0.21 | **0.22** | -0.02 | **-0.06** | -0.17 | **-0.15** | -0.16 | **-0.18** | -0.11 | **-0.07** |
| **Pinot Gris** | Mean | 341 | 349 | 80 | 75 | 122 | 112 | 179 | 168 | 226 | 221 |
|  | (Min, Max) | (328, 360) | (342, 358) | (73, 97) | (69, 81) | (107, 143) | (106, 119) | (163, 200) | (163, 174) | (216, 243) | (219, 224) |
|  | Trend | 0.19 | **0.17** | 0.02 | **-0.07** | -0.17 | **-0.14** | -0.17 | **-0.18** | -0.1 | **-0.07** |
| **Sauvignon Blanc** | Mean | 335 | 342 | 77 | 72 | 124 | 114 | 178 | 167 | 232 | 226 |
|  | (Min, Max) | (323, 354) | (335, 349) | (70, 95) | (66, 79) | (108, 148) | (108, 121) | (161, 200) | (162, 173) | (222, 248) | (224, 230) |
|  | Trend | 0.13 | **0.18** | -0.01 | **-0.07** | -0.2 | **-0.15** | -0.18 | **-0.18** | -0.11 | **-0.07** |
|  |  |  |  |  |  |  |  |  |  |  |  |
| **Monterey** |  | **Chill Completion (DOY)** | | **BUDBREAK (DOY)** | | **BLOOM (DOY)** | | **VERAISON (DOY)** | | **MATURITY (DOY)** | |
|  |  | **1991-2020, obs.** | **2040-2069, RCP4.5** | **1991-2020, obs.** | **2040-2069, RCP4.5** | **1991-2020, obs.** | **2040-2069, RCP4.5** | **1991-2020, obs.** | **2040-2069, RCP4.5** | **1991-2020, obs.** | **2040-2069, RCP4.5** |
| **Cabernet Sauvignon** | Mean | 365 | 380 | 83 | 76 | 143 | 126 | 239 | 215 | 271 | 263 |
|  | (Min, Max) | (343, 424) | (356, 413) | (70, 121) | (68, 88) | (114, 207) | (111, 146) | (181, 365) | (181, 276) | (242, 304) | (245, 302) |
|  | Trend | 0.33 | **0.29** | 0.03 | **-0.11** | -0.28 | **-0.24** | -0.33 | **-0.38** | -0.22 | **-0.22** |
| **Pinot Noir** | Mean | 336 | 346 | 78 | 71 | 136 | 121 | 230 | 206 | 258 | 247 |
|  | (Min, Max) | (322, 373) | (329, 379) | (68, 118) | (64, 83) | (109, 190) | (106, 141) | (177, 357) | (175, 258) | (226, 304) | (228, 289) |
|  | Trend | 0.23 | **0.27** | -0.01 | **-0.09** | -0.28 | **-0.24** | -0.2 | **-0.34** | -0.35 | **-0.21** |
| **Zinfandel** | Mean | 351 | 364 | 84 | 77 | 140 | 125 | 232 | 207 | 262 | 252 |
|  | (Min, Max) | (332, 419) | (343, 401) | (72, 120) | (70, 88) | (115, 189) | (112, 144) | (179, 365) | (179, 253) | (231, 304) | (234, 295) |
|  | Trend | 0.26 | **0.31** | 0.03 | **-0.1** | -0.22 | **-0.22** | -0.25 | **-0.33** | -0.31 | **-0.21** |
| **Chardonnay** | Mean | 358 | 372 | 78 | 72 | 138 | 124 | 225 | 203 | 258 | 247 |
|  | (Min, Max) | (338, 422) | (350, 409) | (69, 115) | (66, 83) | (114, 183) | (112, 143) | (178, 310) | (178, 240) | (226, 304) | (228, 289) |
|  | Trend | 0.32 | **0.31** | 0 | **-0.08** | -0.23 | **-0.2** | -0.18 | **-0.3** | -0.35 | **-0.21** |
| **Pinot Gris** | Mean | 344 | 355 | 82 | 76 | 136 | 122 | 225 | 202 | 253 | 241 |
|  | (Min, Max) | (327, 388) | (337, 388) | (71, 119) | (69, 86) | (113, 182) | (109, 141) | (178, 319) | (177, 242) | (220, 304) | (223, 288) |
|  | Trend | 0.28 | **0.28** | 0.02 | **-0.08** | -0.23 | **-0.2** | -0.19 | **-0.31** | -0.36 | **-0.21** |
| **Sauvignon Blanc** | Mean | 336 | 346 | 79 | 73 | 140 | 125 | 227 | 203 | 258 | 247 |
|  | (Min, Max) | (322, 373) | (329, 379) | (69, 118) | (66, 84) | (115, 187) | (112, 144) | (176, 355) | (176, 246) | (226, 304) | (228, 289) |
|  | Trend | 0.23 | **0.27** | 0 | **-0.09** | -0.24 | **-0.21** | -0.22 | **-0.32** | -0.35 | **-0.21** |
|  |  |  |  |  |  |  |  |  |  |  |  |
| **Paso Robles** |  | **Chill Completion (DOY)** | | **BUDBREAK (DOY)** | | **BLOOM (DOY)** | | **VERAISON (DOY)** | | **MATURITY (DOY)** | |
|  |  | **1991-2020, obs.** | **2040-2069, RCP4.5** | **1991-2020, obs.** | **2040-2069, RCP4.5** | **1991-2020, obs.** | **2040-2069, RCP4.5** | **1991-2020, obs.** | **2040-2069, RCP4.5** | **1991-2020, obs.** | **2040-2069, RCP4.5** |
| **Cabernet Sauvignon** | Mean | 356 | 364 | 86 | 83 | 140 | 129 | 211 | 196 | 259 | 251 |
|  | (Min, Max) | (342, 387) | (352, 386) | (72, 123) | (77, 94) | (113, 179) | (117, 146) | (181, 272) | (180, 221) | (242, 301) | (245, 263) |
|  | Trend | 0.17 | **0.21** | 0.11 | **-0.12** | -0.21 | **-0.18** | -0.24 | **-0.27** | -0.04 | **-0.12** |
| **Pinot Noir** | Mean | 331 | 336 | 80 | 78 | 135 | 125 | 204 | 190 | 242 | 234 |
|  | (Min, Max) | (321, 354) | (328, 351) | (68, 119) | (74, 87) | (109, 172) | (114, 141) | (175, 261) | (175, 214) | (225, 281) | (228, 245) |
|  | Trend | 0.09 | **0.17** | 0.09 | **-0.09** | -0.27 | **-0.19** | -0.23 | **-0.26** | -0.05 | **-0.11** |
| **Zinfandel** | Mean | 344 | 350 | 86 | 84 | 139 | 129 | 206 | 192 | 247 | 239 |
|  | (Min, Max) | (331, 368) | (341, 370) | (73, 121) | (79, 94) | (115, 174) | (118, 144) | (178, 259) | (178, 215) | (231, 291) | (233, 251) |
|  | Trend | 0.18 | **0.18** | 0.1 | **-0.12** | -0.2 | **-0.17** | -0.22 | **-0.25** | -0.05 | **-0.11** |
| **Chardonnay** | Mean | 350 | 357 | 80 | 79 | 137 | 128 | 204 | 191 | 242 | 234 |
|  | (Min, Max) | (337, 378) | (347, 381) | (70, 114) | (75, 86) | (115, 170) | (118, 143) | (178, 248) | (178, 212) | (225, 281) | (228, 245) |
|  | Trend | 0.16 | **0.2** | 0.07 | **-0.08** | -0.21 | **-0.17** | -0.16 | **-0.24** | -0.05 | **-0.11** |
| **Pinot Gris** | Mean | 337 | 343 | 84 | 83 | 135 | 126 | 203 | 190 | 236 | 228 |
|  | (Min, Max) | (326, 363) | (334, 360) | (72, 119) | (78, 92) | (112, 168) | (117, 141) | (176, 248) | (176, 212) | (220, 274) | (222, 240) |
|  | Trend | 0.11 | **0.17** | 0.13 | **-0.1** | -0.22 | **-0.17** | -0.16 | **-0.24** | -0.06 | **-0.11** |
| **Sauvignon Blanc** | Mean | 331 | 336 | 82 | 80 | 138 | 129 | 202 | 189 | 242 | 234 |
|  | (Min, Max) | (321, 354) | (328, 351) | (70, 119) | (75, 88) | (114, 174) | (118, 144) | (175, 251) | (175, 211) | (225, 281) | (228, 245) |
|  | Trend | 0.09 | **0.17** | 0.1 | **-0.09** | -0.2 | **-0.18** | -0.19 | **-0.25** | -0.05 | **-0.11** |
|  |  |  |  |  |  |  |  |  |  |  |  |
| **SLO Coast** |  | **Chill Completion (DOY)** | | **BUDBREAK (DOY)** | | **BLOOM (DOY)** | | **VERAISON (DOY)** | | **MATURITY (DOY)** | |
|  |  | **1991-2020, obs.** | **2040-2069, RCP4.5** | **1991-2020, obs.** | **2040-2069, RCP4.5** | **1991-2020, obs.** | **2040-2069, RCP4.5** | **1991-2020, obs.** | **2040-2069, RCP4.5** | **1991-2020, obs.** | **2040-2069, RCP4.5** |
| **Cabernet Sauvignon** | Mean | 374 | 391 | 82 | 80 | 149 | 133 | 260 | 235 | 280 | 270 |
|  | (Min, Max) | (346, 424) | (362, 417) | (69, 125) | (76, 94) | (106, 220) | (117, 156) | (195, 365) | (193, 294) | (246, 304) | (248, 304) |
|  | Trend | 0.33 | **0.29** | 0.04 | **-0.09** | -0.21 | **-0.25** | -0.26 | **-0.41** | 0 | **-0.25** |
| **Pinot Noir** | Mean | 342 | 355 | 77 | 76 | 140 | 127 | 250 | 223 | 268 | 253 |
|  | (Min, Max) | (323, 403) | (334, 386) | (64, 120) | (72, 87) | (103, 203) | (114, 145) | (188, 364) | (186, 278) | (229, 304) | (231, 295) |
|  | Trend | 0.26 | **0.31** | -0.01 | **-0.06** | -0.23 | **-0.23** | -0.2 | **-0.37** | -0.12 | **-0.26** |
| **Zinfandel** | Mean | 358 | 375 | 83 | 82 | 144 | 132 | 249 | 223 | 272 | 259 |
|  | (Min, Max) | (334, 422) | (349, 408) | (70, 122) | (77, 94) | (108, 200) | (118, 149) | (190, 362) | (189, 273) | (235, 304) | (236, 298) |
|  | Trend | 0.27 | **0.33** | 0.05 | **-0.08** | -0.14 | **-0.21** | -0.39 | **-0.34** | -0.08 | **-0.27** |
| **Chardonnay** | Mean | 366 | 384 | 77 | 77 | 141 | 131 | 240 | 217 | 268 | 253 |
|  | (Min, Max) | (340, 424) | (355, 410) | (68, 115) | (74, 86) | (109, 191) | (119, 145) | (188, 361) | (187, 254) | (229, 304) | (231, 295) |
|  | Trend | 0.3 | **0.32** | 0.01 | **-0.06** | -0.16 | **-0.19** | -0.34 | **-0.3** | -0.12 | **-0.26** |
| **Pinot Gris** | Mean | 350 | 365 | 81 | 80 | 139 | 128 | 241 | 217 | 264 | 248 |
|  | (Min, Max) | (328, 421) | (342, 396) | (70, 120) | (76, 92) | (107, 188) | (117, 143) | (186, 362) | (186, 257) | (224, 304) | (225, 292) |
|  | Trend | 0.28 | **0.35** | 0.03 | **-0.07** | -0.17 | **-0.2** | -0.32 | **-0.31** | -0.07 | **-0.26** |
| **Sauvignon Blanc** | Mean | 342 | 355 | 78 | 77 | 143 | 132 | 243 | 218 | 268 | 253 |
|  | (Min, Max) | (323, 403) | (334, 386) | (68, 120) | (74, 88) | (108, 198) | (118, 148) | (185, 365) | (185, 264) | (229, 304) | (231, 295) |
|  | Trend | 0.26 | **0.31** | 0 | **-0.06** | -0.15 | **-0.21** | -0.35 | **-0.33** | -0.12 | **-0.26** |
|  |  |  |  |  |  |  |  |  |  |  |  |
| **Santa Ynez Valley** |  | **Chill Completion (DOY)** | | **BUDBREAK (DOY)** | | **BLOOM (DOY)** | | **VERAISON (DOY)** | | **MATURITY (DOY)** | |
|  |  | **1991-2020, obs.** | **2040-2069, RCP4.5** | **1991-2020, obs.** | **2040-2069, RCP4.5** | **1991-2020, obs.** | **2040-2069, RCP4.5** | **1991-2020, obs.** | **2040-2069, RCP4.5** | **1991-2020, obs.** | **2040-2069, RCP4.5** |
| **Cabernet Sauvignon** | Mean | 375 | 392 | 78 | 77 | 131 | 121 | 221 | 202 | 264 | 253 |
|  | (Min, Max) | (351, 424) | (373, 418) | (68, 105) | (73, 82) | (103, 166) | (109, 132) | (180, 291) | (180, 223) | (241, 304) | (244, 262) |
|  | Trend | 0.28 | **0.25** | -0.01 | **-0.08** | -0.27 | **-0.24** | -0.14 | **-0.32** | -0.13 | **-0.15** |
| **Pinot Noir** | Mean | 342 | 355 | 73 | 73 | 125 | 116 | 212 | 194 | 246 | 236 |
|  | (Min, Max) | (324, 390) | (340, 393) | (62, 97) | (70, 78) | (99, 162) | (106, 127) | (174, 273) | (174, 213) | (225, 290) | (227, 245) |
|  | Trend | 0.17 | **0.3** | -0.04 | **-0.06** | -0.18 | **-0.24** | -0.17 | **-0.31** | -0.11 | **-0.15** |
| **Zinfandel** | Mean | 359 | 375 | 78 | 78 | 130 | 121 | 213 | 196 | 252 | 242 |
|  | (Min, Max) | (339, 422) | (356, 415) | (70, 104) | (74, 83) | (106, 163) | (111, 131) | (177, 268) | (177, 214) | (230, 296) | (233, 251) |
|  | Trend | 0.2 | **0.33** | 0.01 | **-0.07** | -0.21 | **-0.21** | -0.18 | **-0.29** | -0.12 | **-0.14** |
| **Chardonnay** | Mean | 367 | 384 | 73 | 74 | 129 | 121 | 208 | 194 | 246 | 236 |
|  | (Min, Max) | (345, 423) | (364, 417) | (66, 91) | (71, 79) | (106, 160) | (112, 130) | (177, 255) | (177, 209) | (225, 290) | (227, 245) |
|  | Trend | 0.23 | **0.29** | -0.03 | **-0.05** | -0.17 | **-0.2** | -0.15 | **-0.27** | -0.11 | **-0.15** |
| **Pinot Gris** | Mean | 350 | 365 | 77 | 77 | 127 | 119 | 208 | 193 | 241 | 230 |
|  | (Min, Max) | (331, 419) | (348, 404) | (69, 98) | (74, 82) | (104, 157) | (110, 128) | (175, 257) | (175, 209) | (219, 283) | (221, 240) |
|  | Trend | 0.15 | **0.34** | -0.02 | **-0.07** | -0.16 | **-0.2** | -0.13 | **-0.27** | -0.1 | **-0.15** |
| **Sauvignon Blanc** | Mean | 342 | 355 | 74 | 75 | 130 | 121 | 208 | 193 | 246 | 236 |
|  | (Min, Max) | (324, 390) | (340, 393) | (65, 96) | (71, 80) | (106, 163) | (111, 131) | (174, 260) | (174, 209) | (225, 290) | (227, 245) |
|  | Trend | 0.17 | **0.3** | -0.04 | **-0.06** | -0.18 | **-0.21** | -0.14 | **-0.28** | -0.11 | **-0.15** |
|  |  |  |  |  |  |  |  |  |  |  |  |

**Supplemental Table 2.** Correlation and Significance Analysis between Metrics used in this study and Annual Mean Temperature and Averaged DEM in Various Time Periods.

|  | | | Tmean_ann | | △ Tmean_ann | |  |  |
| --- | --- | --- | --- | --- | --- | --- | --- | --- |
|  |  |  | Current | | RCP 4.5 – Current | | DEM | |
|  |  |  | r | p | r | p | r | p |
| Variety-specific metrics | Current | Chill | 0.15 | 0.65 |  |  | -0.47 | 0.12 |
|  |  | Budburst | **-0.59** | 0.04 |  |  | **0.69** | 0.01 |
|  |  | Flowering | **-0.95** | 0.00 |  |  | 0.46 | 0.14 |
|  |  | Veraison | **-0.88** | 0.00 |  |  | -0.02 | 0.95 |
|  |  | Maturity | **-0.90** | 0.00 |  |  | 0.17 | 0.59 |
|  | RCP4.5-Current | △ Chill | -0.20 | 0.53 | **-0.65** | 0.02 | -0.46 | 0.14 |
|  |  | △ Budburst | 0.33 | 0.29 | **-0.57** | 0.05 | -0.18 | 0.57 |
|  |  | △ Flowering | **0.73** | 0.01 | -0.01 | 0.97 | 0.07 | 0.83 |
|  |  | △ Veraison | **0.75** | 0.01 | **0.59** | 0.04 | 0.31 | 0.33 |
|  |  | △ Maturity | **0.87** | 0.00 | 0.48 | 0.11 | -0.14 | 0.67 |
| General metrics | Current | GDD | **0.89** | 0.00 |  |  | -0.01 | 0.98 |
|  |  | DD_c_ | -0.12 | 0.70 |  |  | 0.52 | 0.09 |
|  |  | LSF | 0.11 | 0.74 |  |  | **0.66** | 0.02 |
|  |  | FFF | -0.10 | 0.75 |  |  | -0.39 | 0.22 |
|  |  | FFS | -0.27 | 0.39 |  |  | -0.56 | 0.06 |
|  |  | HD35 | **0.77** | 0.00 |  |  | -0.05 | 0.89 |
|  |  | HW | 0.11 | 0.74 |  |  | 0.26 | 0.41 |
|  |  | DTR | 0.43 | 0.17 |  |  | 0.13 | 0.70 |
|  |  | DTR20 | 0.21 | 0.51 |  |  | 0.08 | 0.81 |
|  |  | Pr_ex_ | **-0.58** | 0.05 |  |  | 0.52 | 0.08 |
|  |  | Pr_acc_ | **-0.75** | 0.01 |  |  | 0.35 | 0.27 |
|  |  | ET_c_ | **0.61** | 0.03 |  |  | 0.07 | 0.83 |
|  |  | H_ini_ | -0.15 | 0.65 |  |  | **0.83** | 0.00 |
|  |  | FDD | -0.05 | 0.88 |  |  | **0.77** | 0.00 |
|  | RCP4.5-Current | △ GDD | 0.52 | 0.09 | **0.96** | 0.00 | -0.05 | 0.87 |
|  |  | △ DD_c_ | 0.20 | 0.54 | -0.03 | 0.91 | **0.57** | 0.05 |
|  |  | △ LSF | -0.26 | 0.41 | -0.32 | 0.30 | -0.45 | 0.14 |
|  |  | △ FFF | -0.36 | 0.26 | -0.44 | 0.15 | -0.26 | 0.42 |
|  |  | △ FFS | 0.34 | 0.28 | 0.42 | 0.17 | 0.34 | 0.28 |
|  |  | △ HD35 | **0.68** | 0.02 | **0.81** | 0.00 | 0.27 | 0.40 |
|  |  | △ HW | 0.48 | 0.11 | **0.81** | 0.00 | 0.38 | 0.22 |
|  |  | △ DTR | -0.07 | 0.82 | **0.59** | 0.05 | **0.60** | 0.04 |
|  |  | △ DTR20 | 0.17 | 0.61 | **0.62** | 0.03 | **0.63** | 0.03 |
|  |  | △ Pr_ex_ | -0.27 | 0.39 | 0.04 | 0.90 | 0.22 | 0.48 |
|  |  | △ Pr_acc_ | **-0.73** | 0.01 | -0.15 | 0.64 | 0.08 | 0.81 |
|  |  | △ ET_c_ | 0.09 | 0.79 | -0.10 | 0.75 | -0.51 | 0.09 |
|  |  | △ H_ini_ | 0.15 | 0.65 | -0.44 | 0.15 | **-0.83** | 0.00 |
|  |  | △ FDD | 0.09 | 0.77 | -0.19 | 0.56 | **-0.74** | 0.01 |


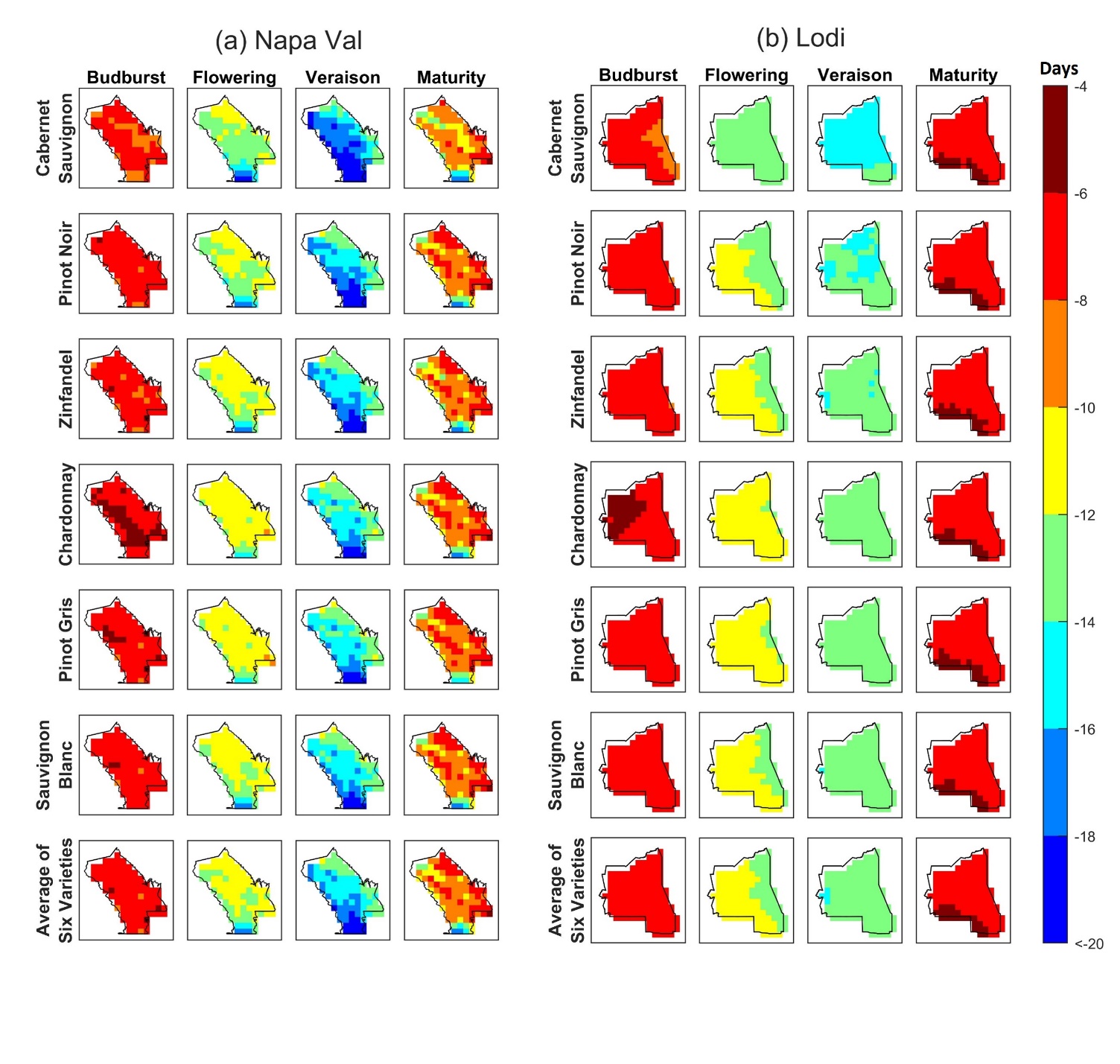


**Supplemental Figure 1.** Changes in the timing of four phenology phases for six winegrape varieties over the topographically heterogeneous (a) Napa Valley and the homogeneous (b) Lodi AVAs. Change is calculated as the 2040-2069 climatological average day of year under RCP 4.5 minus the 1991-2020 observed climatological average day of year. The average change across all six varieties is also shown. Although Supplemental Figure 6b (Lodi) appears to show an east-west divide in flowering day of year for some varieties, this is an artifact of the visualization and the average change in DOY varies by <4 days across the AVA.

**Supplemental Table 3.** The mean, range, and 30-year trend of 14 agroclimate metrics over 12 AVAs under the contemporary (1991-2020) and future (2040-2069, RCP 4.5) periods. Statistically significant trends are in bold.

|  |  | **GDD** | | **DDc** | | **LSF** | | **FFF** | | **FFS** | | **HD35** | | **HW** | | **DTR** | | **DTR20** | | **Pr_ex_** | | **Pr_acc_** | | **ETc** | |
| --- | --- | --- | --- | --- | --- | --- | --- | --- | --- | --- | --- | --- | --- | --- | --- | --- | --- | --- | --- | --- | --- | --- | --- | --- | --- |
|  |  | **Cont.** | **RCP4.5** | **Cont.** | **RCP4.5** | **Cont.** | **RCP4.5** | **Cont.** | **RCP4.5** | **Cont.** | **RCP4.5** | **Cont.** | **RCP4.5** | **Cont.** | **RCP4.5** | **Cont.** | **RCP4.5** | **Cont.** | **RCP4.5** | **Cont.** | **RCP4.5** | **Cont.** | **RCP4.5** | **Cont.** | **RCP4.5** |
| **West Sonoma Coast** | **Mean** | 1282 | 1651 | 1704 | 1250 | 29 | 33 | 340 | 350 | 350 | 361 | 1 | 0 | 0 | 0 | 14 | 14 | 1 | 0 | 6 | 6 | 645 | 701 | 474 | 515 |
|  | **(Min, Max)** | (540, 1928) | (1034, 2005) | (1006, 2168) | (981, 1495) | (1, 68) | (1, 117) | (303, 365) | (292, 363) | (249, 365) | (349, 365) | (0, 12) | (0, 4) | (0, 1) | (0, 0) | (9, 18) | (10, 17) | (0, 34) | (0, 5) | (1, 16) | (3, 10) | (59, 1815) | (430, 1207) | (329, 595) | (394, 603) |
|  | **Trend** | 2.96 | **4.79** | -5.76 | **-8.48** | 0.23 | -0.19 | -0.72 | -0.13 | **-1.14** | 0.03 | **0.01** | **0.01** | 0 | 0 | -0.02 | 0 | **0.06** | 0 | -0.06 | -0.01 | -5.83 | 3.28 | 0.53 | **0.32** |
| **El Dorado** | **Mean** | 2080 | 2492 | 2024 | 1728 | 76 | 58 | 327 | 333 | 252 | 286 | 23 | 55 | 0 | 2 | 16 | 17 | 3 | 11 | 7 | 7 | 492 | 513 | 550 | 580 |
|  | **(Min, Max)** | (1413, 2669) | (2037, 2822) | (1271, 2573) | (1264, 2229) | (1, 154) | (22, 106) | (278, 363) | (307, 349) | (142, 362) | (201, 345) | (0, 59) | (25, 80) | (0, 3) | (0, 4) | (11, 19) | (14, 18) | (0, 33) | (1, 27) | (1, 19) | (3, 12) | (71, 1701) | (309, 804) | (467, 638) | (527, 639) |
|  | **Trend** | 5.49 | **5.95** | **-8.74** | **-7.04** | -0.24 | **-0.29** | 0.46 | **0.17** | 0.55 | **0.71** | 0.03 | **0.33** | 0 | **0.02** | **-0.08** | 0 | **-0.13** | -0.02 | -0.1 | -0.01 | -1.33 | 1.95 | 0.6 | **0.43** |
| **Livermore**  **Valley** | **Mean** | 1919 | 2310 | 1615 | 1206 | 33 | 33 | 342 | 350 | 329 | 351 | 11 | 24 | 0 | 0 | 15 | 15 | 5 | 5 | 3 | 3 | 258 | 267 | 575 | 617 |
|  | **(Min, Max)** | (1350, 2521) | (1893, 2582) | (824, 2132) | (834, 1480) | (1, 111) | (2, 104) | (317, 365) | (335, 362) | (239, 365) | (326, 365) | (0, 33) | (5, 44) | (0, 1) | (0, 1) | (10, 18) | (12, 18) | (0, 28) | (0, 17) | (0, 12) | (1, 5) | (34, 726) | (139, 448) | (473, 679) | (563, 677) |
|  | **Trend** | 5.08 | **5.76** | -6.72 | **-8.5** | -0.09 | 0.00 | 0.20 | -0.09 | -0.43 | **0.25** | **0.27** | **0.21** | 0 | 0 | -0.02 | 0 | 0.05 | -0.02 | -0.05 | -0.01 | -1.52 | 1.32 | **0.98** | **0.41** |
| **Lodi** | **Mean** | 2342 | 2767 | 1669 | 1283 | 52 | 38 | 331 | 339 | 286 | 321 | 37 | 68 | 0 | 1 | 17 | 18 | 21 | 21 | 3 | 3 | 217 | 230 | 603 | 640 |
|  | **(Min, Max)** | (1943, 2719) | (2546, 2946) | (1140, 2033) | (1083, 1511) | (1, 109) | (19, 60) | (300, 365) | (323, 353) | (201, 365) | (286, 355) | (16, 66) | (51, 88) | (0, 2) | (0, 2) | (14, 20) | (16, 19) | (1, 55) | (9, 36) | (0, 13) | (1, 7) | (36, 597) | (123, 356) | (509, 745) | (593, 714) |
|  | **Trend** | 3.87 | **5.87** | -4.28 | **-8.74** | -1.01 | -0.12 | **0.72** | -0.03 | **1.64** | **0.46** | 0.25 | **0.36** | 0 | **0.01** | 0 | 0 | 0.02 | -0.03 | -0.07 | -0.02 | -1.3 | 0.94 | **1.23** | **0.46** |
| **Madera** | **Mean** | 2596 | 3014 | 1722 | 1354 | 45 | 36 | 332 | 340 | 293 | 324 | 61 | 93 | 0 | 2 | 18 | 18 | 20 | 22 | 2 | 2 | 126 | 131 | 671 | 718 |
|  | **(Min, Max)** | (2155, 3042) | (2817, 3257) | (1229, 2116) | (1116, 1581) | (1, 111) | (22, 57) | (306, 365) | (330, 349) | (218, 364) | (295, 344) | (20, 107) | (75, 120) | (0, 2) | (1, 3) | (16, 20) | (17, 19) | (0, 52) | (12, 45) | (0, 9) | (1, 3) | (23, 364) | (71, 195) | (559, 789) | (656, 775) |
|  | **Trend** | **6.1** | **4.98** | -7.39 | **-7.44** | 0.03 | 0.01 | 0.44 | 0.02 | 0.66 | **0.34** | **0.76** | **0.39** | 0 | **0.02** | 0 | 0 | -0.06 | -0.01 | -0.02 | 0 | -0.34 | 0.38 | **1.78** | **0.4** |
| **Mendocino** | **Mean** | 1786 | 2142 | 1960 | 1619 | 66 | 49 | 326 | 338 | 267 | 312 | 20 | 36 | 0 | 1 | 18 | 18 | 33 | 33 | 6 | 6 | 559 | 603 | 667 | 714 |
|  | **(Min, Max)** | (1071, 2364) | (1581, 2443) | (1145, 2534) | (1189, 2055) | (1, 136) | (4, 87) | (259, 365) | (317, 361) | (151, 365) | (236, 359) | (0, 70) | (2, 64) | (0, 3) | (0, 3) | (9, 23) | (11, 21) | (0, 84) | (0, 66) | (1, 17) | (4, 10) | (45, 1292) | (395, 902) | (443, 867) | (533, 840) |
|  | **Trend** | **8.17** | **5.46** | -6.33 | **-7.39** | 0.01 | **-0.35** | **0.55** | 0.00 | 0.03 | **0.77** | **0.4** | **0.22** | 0 | 0.01 | -0.02 | 0 | -0.11 | -0.03 | -0.07 | -0.01 | -4.16 | 3 | **2.36** | **0.45** |
| **Monterey** | **Mean** | 1601 | 1964 | 1487 | 1146 | 54 | 41 | 329 | 343 | 303 | 328 | 12 | 21 | 0 | 0 | 16 | 16 | 20 | 21 | 2 | 2 | 180 | 187 | 506 | 549 |
|  | **(Min, Max)** | (846, 2449) | (1342, 2715) | (532, 2049) | (525, 1700) | (1, 154) | (1, 98) | (253, 365) | (314, 365) | (124, 365) | (221, 365) | (0, 79) | (0, 95) | (0, 2) | (0, 2) | (7, 25) | (8, 24) | (0, 91) | (0, 84) | (0, 8) | (1, 4) | (5, 617) | (95, 355) | (328, 708) | (386, 713) |
|  | **Trend** | **6.12** | **5.31** | -6.28 | **-7.09** | -0.11 | -0.64 | 0.39 | -0.03 | -0.39 | **0.57** | **0.2** | **0.13** | 0 | **0.01** | 0 | 0 | 0.1 | 0 | -0.02 | 0 | 0.1 | 0.74 | **1.53** | **0.33** |
| **Napa Valley** | **Mean** | 1978 | 2367 | 1739 | 1377 | 49 | 37 | 337 | 345 | 306 | 340 | 18 | 38 | 0 | 1 | 17 | 17 | 14 | 16 | 5 | 4 | 435 | 466 | 581 | 623 |
|  | **(Min, Max)** | (1309, 2649) | (1915, 2835) | (999, 2303) | (931, 1814) | (1, 118) | (2, 105) | (296, 365) | (326, 363) | (188, 365) | (283, 365) | (0, 62) | (4, 80) | (0, 3) | (0, 3) | (11, 21) | (13, 20) | (0, 73) | (0, 54) | (0, 14) | (2, 8) | (36, 1374) | (232, 802) | (430, 760) | (515, 751) |
|  | **Trend** | 4.3 | **5.79** | -7.21 | **-8.43** | 0.25 | -0.24 | 0.19 | 0.03 | -0.19 | **0.52** | 0.22 | **0.28** | 0 | **0.01** | 0 | 0 | 0.14 | -0.04 | -0.08 | -0.01 | -3.21 | 2.16 | **1.33** | **0.48** |
| **Paso Robles** | **Mean** | 1971 | 2335 | 1765 | 1498 | 75 | 58 | 318 | 332 | 246 | 283 | 40 | 63 | 0 | 0 | 21 | 21 | 64 | 65 | 2 | 2 | 207 | 217 | 617 | 658 |
|  | **(Min, Max)** | (1289, 2655) | (1809, 2753) | (1034, 2146) | (1065, 1838) | (3, 157) | (4, 111) | (258, 362) | (302, 361) | (118, 365) | (199, 362) | (0, 81) | (5, 95) | (0, 1) | (0, 2) | (17, 26) | (18, 25) | (5, 92) | (21, 89) | (0, 11) | (1, 6) | (8, 1005) | (73, 500) | (523, 713) | (593, 726) |
|  | **Trend** | **6.46** | **5.25** | -3.97 | **-5.61** | 0.35 | **-0.50** | 0.18 | **0.21** | -0.71 | **0.94** | **0.71** | **0.35** | 0 | **0.01** | -0.02 | -0.01 | -0.32 | -0.04 | -0.02 | 0 | -0.71 | 0.54 | **1.29** | **0.35** |
| **Russian River Valley** | **Mean** | 1662 | 2050 | 1703 | 1334 | 32 | 29 | 336 | 349 | 325 | 352 | 5 | 10 | 0 | 0 | 17 | 17 | 15 | 10 | 5 | 5 | 521 | 564 | 544 | 588 |
|  | **(Min, Max)** | (1275, 2162) | (1770, 2428) | (978, 2027) | (1028, 1594) | (1, 114) | (3, 104) | (301, 365) | (332, 363) | (225, 365) | (331, 365) | (0, 29) | (0, 43) | (0, 1) | (0, 1) | (14, 21) | (15, 19) | (0, 59) | (0, 25) | (0, 14) | (3, 8) | (46, 1370) | (332, 914) | (471, 667) | (531, 664) |
|  | **Trend** | 2.72 | **5.47** | -5.05 | **-7.7** | 0.49 | 0.05 | -0.38 | -0.05 | **-1.56** | **0.20** | **0.32** | **0.13** | 0 | 0 | 0.02 | 0 | **0.77** | -0.03 | -0.06 | -0.01 | -4.38 | 2.47 | **1.13** | **0.38** |
| **Santa Ynez Valley** | **Mean** | 1853 | 2196 | 1253 | 920 | 38 | 34 | 341 | 349 | 326 | 349 | 9 | 17 | 0 | 0 | 17 | 17 | 14 | 10 | 2 | 2 | 227 | 241 | 562 | 602 |
|  | **(Min, Max)** | (1273, 2608) | (1829, 2771) | (435, 1689) | (402, 1273) | (1, 154) | (1, 97) | (303, 365) | (320, 363) | (187, 365) | (307, 365) | (0, 60) | (0, 69) | (0, 2) | (0, 1) | (11, 23) | (12, 21) | (0, 89) | (0, 63) | (0, 9) | (1, 4) | (20, 803) | (135, 391) | (444, 663) | (543, 664) |
|  | **Trend** | **7.78** | **5.3** | -3.12 | **-6.52** | 0.34 | -0.29 | 0.05 | 0.03 | -0.98 | **0.39** | **0.43** | **0.15** | 0 | 0 | 0.01 | 0 | **0.36** | -0.01 | -0.01 | -0.01 | -0.49 | 0.08 | **1.51** | **0.27** |
| **SLO Coast** | **Mean** | 1388 | 1720 | 1299 | 937 | 32 | 30 | 344 | 353 | 346 | 358 | 1 | 1 | 0 | 0 | 13 | 13 | 2 | 1 | 2 | 3 | 277 | 296 | 463 | 498 |
|  | **(Min, Max)** | (604, 2227) | (1215, 2371) | (439, 1949) | (430, 1531) | (1, 119) | (1, 98) | (284, 365) | (321, 365) | (172, 365) | (293, 365) | (0, 30) | (0, 29) | (0, 6) | (0, 3) | (5, 20) | (8, 19) | (0, 50) | (0, 30) | (0, 11) | (1, 7) | (15, 1788) | (137, 751) | (317, 638) | (388, 640) |
|  | **Trend** | 5.89 | **4.63** | -6.3 | **-6.88** | 0.38 | 0.38 | 0.23 | -0.12 | **-0.92** | **0.15** | **0.04** | **0.02** | 0 | 0 | -0.01 | 0 | **0.09** | 0 | -0.03 | 0 | -0.76 | 0.78 | **0.87** | **0.26** |


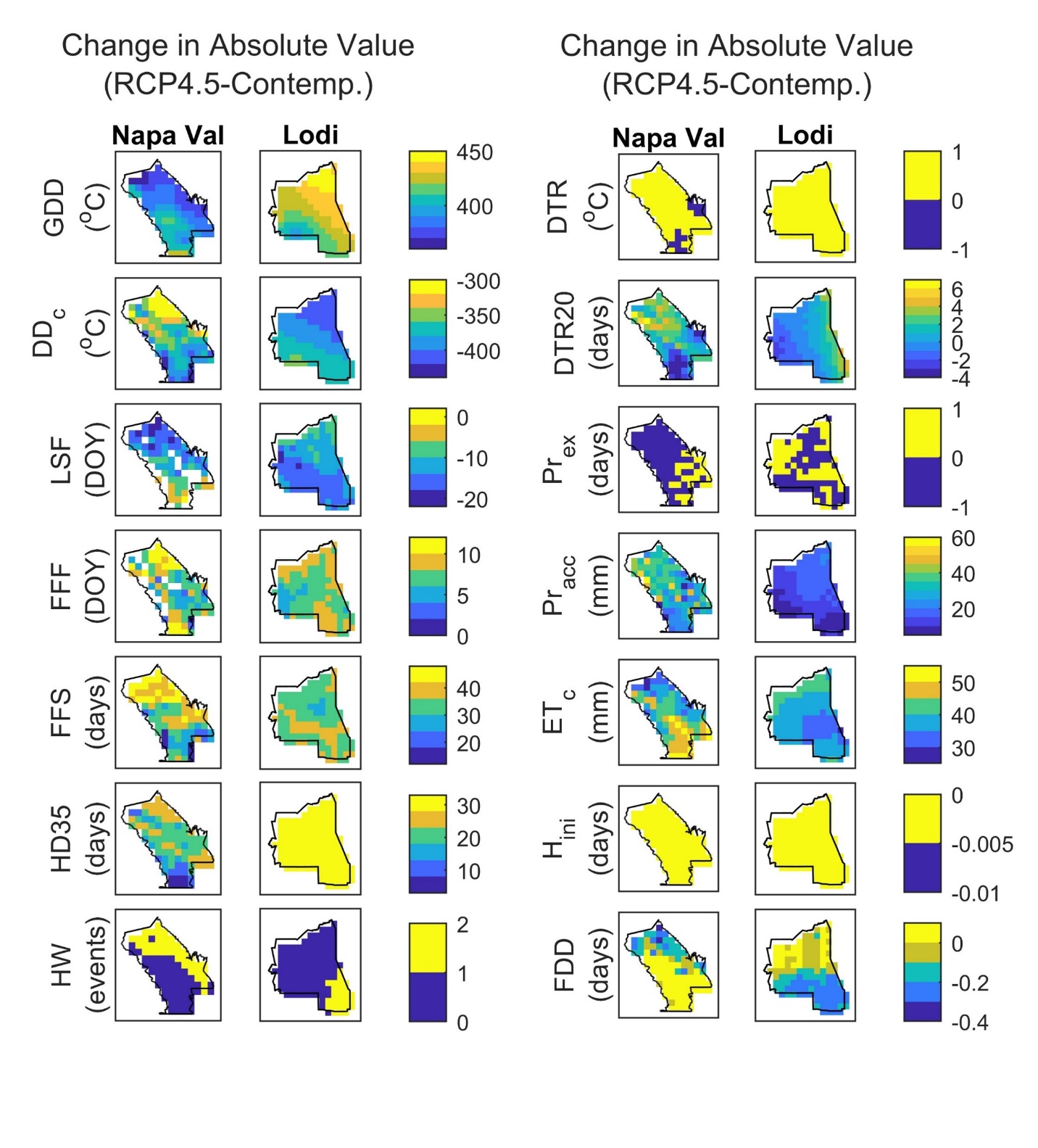


**Supplemental Figure 2.** Changes in 14 viticulturally-important agroclimate metrics over the topographically heterogeneous Napa Valley (left column, both panels) and topographically homogenous Lodi (right column, both panels ) AVAs. Changes are calculated as the 2040-2069 projected climatological value under RCP 4.5 minus the 1991-2020 observed climatological value.
